# AMERICCAASS Registry: A Contemporary View of Heart Failure in the Americas

**DOI:** 10.1161/JAHA.125.047844

**Published:** 2026-08-27

**Authors:** Sebastián Seni‐Molina, Juan Pablo Arango‐Ibanez, Valentina Villegas‐Céspedes, Andrea Valencia‐Orozco, Hoover O. León‐Giraldo, Yudmila Borges, Elizabeth Sellén Sanchén, Mailyn Vilaú Jiménez, Julian Lugo‐Peña, Gustavo Andres Ortega Ramirez, Luis Roberto Lema, Leandro Parrilla, Gerardo Chazzin, Elirub Rojas Gimon, Claudine Judith Coronel Mariño, Pablo Jose Gonzalez Velasquez, Manuel Hipólito Chaple La Hoz, Alfa Kenia Fernandez, Miguel Eduardo Azañedo Velásquez, Clemente Barron Magdaleno, Esteban Zavaleta Monestel, Juan Carlos Henríquez Bonilla, Maximiliano Pereda, Lauren Cooper, Daniel Quesada‐Chaves, Eduardo R. Perna, Víctor Rossel, Mario Speranza, Mark H. Drazner, Walter Alarco, Alexander Romero‐Guerra, Juan Esteban Gómez‐Mesa, the AMERICCAASS Research Group, Juan Esteban Gómez‐Mesa, Eduardo R. Perna, Víctor Rossel, Daniel Quesada‐Chaves, Mario Speranza, Mark H. Drazner, Walter Alarco, Alexander Romero‐Guerra, Sebastián Seni‐Molina, Beder Gustavo Farez, Cristian Núñez‐Carrizo, Norberto Raúl Bornancini, Elda Lorena Brocal Ocampo, Leandro Parrilla, Daniela García Brasca, Juan Pablo Allende, Ana Virginia Figueroa, Luis Guillermo Muñoz, Juan Pablo Escalante, Eduardo R. Perna, Domingo César Azcona, Alberto A. Fernández, Adrián Lescano, Luis Roberto Lema, José René Romano, Orlando David Fernández‐Flores, Juan Justiniano Encina, Víctor Rossel, Julián Lugo‐Peña, Gustavo Andrés Ortega Ramírez, Erika María Martínez Carreño, Alex Rivera‐Toquica, Sebastián Campbell‐Quintero, Álvaro Mauricio Quintero‐Ossa, Luis E. Echeverría, María Juliana Rodríguez‐González, Andrés Felipe Buitrago Sandoval, Juan Esteban Gómez‐Mesa, Guillermo Trout‐Guardiola, Gustavo Adolfo Moreno Silgado, Carlos Hernán Calderón Franco, Erwin Eladio Silva Cardona, Germán Camilo Giraldo‐González, Juan Alberto Cerón Benavides, Alexis José Bastardo López, Esteban Zavaleta Monestel, Andrés Ulate Retana, Daniel Quesada‐Chaves, Aymé Alberna Cardoso, Tessa Negrín Valdés, Migdalia Cristina Cóbas Pérez, Maité Goberna Rocha, José Carlos Acosta Abad, Sucel Lázara Figueredo Carrazana, José Carlos López Martín, Mailyn Vilaú Jiménez, Annia María Carrero‐Vásquez, Maikel Santos Medina, Sandra Companioni Montero, Yudmila Borges, Elizabeth Sellén Sanchén, Tahimí Chacón Quesada, Yilian Herrera Domínguez, Elién García Sánchez, Raciel Victores Chacón, Harold Segundo Cedeño Macías, Fausto Bernardo Heredia Villacreses, Manuel Hipólito Chaple La Hoz, Jorge Gustavo Castro Daul, Ricardo Mármol Alvear, David Alejandro Puga Bermúdez, Marlon Patricio Aguirre Espinosa, Nadia Sánchez, Leonardo Bermúdez, Freddy Pow‐Chon‐Long, María Eugenia Menjívar‐De Ramos, Silvia Carolina Lazo‐Majano, Lauren Cooper, Elizabeth Ashley Hardin, Milton Lubeck Herrera, Luis Arturo Rodríguez, Mádelyn Raquel Valle‐Ramos, Pablo José González Velásquez, Sergio Roberto Fausto Ovando, Emilio Samael Peralta‐López, Emilio Samael Peralta‐López, Felix Nunura Arrese, Eduardo Heberto Herrera Garza, Jesús Eduardo Sánchez Hernández, José Benito Álvarez Mosquera, Eufracino Sandoval Rodríguez, Amada Álvarez‐Sangabriel, Clemente Barron Magdaleno, Luis Carlos Pérez Plata, Nilda Espinola‐Zavaleta, Pablo Hurtado Nuñez, Gabriel Frago, Claudine Judith Coronel Mariño, Guillermo Oviedo‐Pereira, Javier Galeano Figueredo, Luz Teresa Cabral‐Gueyraud, Miguel A. Quintana, Ida Fabiola Rodríguez‐Caballero, Sergio Gabriel Vergara, María Paniagua, Miguel Eduardo Azañedo Velásquez, Guillermo Alberto Arana Morales, Carlos Demetrio Renato Pacheco Bancayan, Luis Felipe Anhuaman‐Atoche, Paola Gissela Oliver Rengifo, Juan Carlos More Terán, Ruddy Miguel García‐Safadit, Sarah Raquel Marte‐Arias, Alfa Kenia Fernández, Licurgo Jacob Cruz‐Díaz, Kwame van der Hilst, Gabriela Ormaechea‐Gorricho, Maximiliano Pereda, Raquel E González‐Hormostay, Ximena Patricia Madrid Viteri, Luis Díaz Espinoza, Eglee Castillo González, Elirub Rojas Gimon, Maite Alexandra González Fuentes, Antonella A. Ferrer, Gerardo Chazzin, Niurka Pereira Velasco, José Jesús Marval Saud

**Affiliations:** ^1^ Centro de Investigaciones Clínicas (CIC), Fundación Valle del Lili Cali Colombia; ^2^ Facultad de Ciencias de la Salud Universidad Icesi Cali Colombia; ^3^ Departamento de Cardiología Instituto de Cardiología y Cirugía Cardiovascular La Habana Cuba; ^4^ Departamento de Cardiología Hospital Manuel Ascunce Domenech Camagüey Cuba; ^5^ Departamento de Cardiología Hospital Provincial Clínico Quirúrgico Abel Santamaría Pinar del Río Cuba; ^6^ Departamento de Cardiología Clínica del Occidente Bogotá Colombia; ^7^ Departamento de Cardiología Fundación Cardioinfantil—Instituto de Cardiología Bogotá Colombia; ^8^ Departamento de Falla Cardiaca Clínica General del Norte Barranquilla Colombia; ^9^ Departamento de Hipertensión Pulmonar Instituto Modelo de Cardiología Privado SRL Córdoba Argentina; ^10^ Unidad Coronaria, Hospital General de Agudos Carlos G. Durand Buenos Aires Argentina; ^11^ Departamento de Cardiología UNICOR/FUNVIC Valencia Venezuela; ^12^ Departamento de Cardiología Cardiología Tropical Anzoátegui Lechería Anzoátegui Venezuela; ^13^ Departamento de Cardiología Clínica Clínicor Concepción Paraguay; ^14^ Departamento de Cardiología Hospital San Juan de Dios Ciudad de Guatemala Guatemala; ^15^ Departamento de Cardiología Centro Médico AMEDS Manta Ecuador; ^16^ Programa de Medicina Pontificia Universidad Católica del Ecuador Manabí Ecuador; ^17^ Departamento de Cardiología Hospital Metropolitano de Santiago (HOMS) Santiago de los Caballeros Dominican Republic; ^18^ Unidad de Insuficiencia Cardiaca Instituto Cardiovascular Clinicor Norte Lima Peru; ^19^ Departamento de Cardiología Instituto Nacional de Ciencias Médicas y Nutrición Ciudad de México Mexico; ^20^ Departamento de Investigación Hospital Clínica Bíblica San José Costa Rica; ^21^ Departamento de Cardiología Clínica del Corazón Sinusal San Miguel El Salvador; ^22^ Departamento de Cardiología Médica Uruguaya Montevideo Uruguay; ^23^ Cardiology Department North Shore University Hospital Manhasset; ^24^ Servicio de Cardiología Hospital San Vicente de Paul Heredia Costa Rica; ^25^ Unidad de Falla Cardíaca e Hipertensión Pulmonar Instituto De Cardiología J. F. Cabral Corrientes Argentina; ^26^ Servicio de Cardiología Hospital del Salvador e Instituto Nacional del Tórax Santiago Chile; ^27^ Facultad de Medicina Universidad de Chile Santiago Chile; ^28^ Departamento de Cardiología Hospital Clínica Bíblica San José Costa Rica; ^29^ Division of Cardiology, Department of Internal Medicine University of Texas Southwestern Medical Center Dallas; ^30^ Servicio de Cardiología Instituto Nacional Cardiovascular INCOR Lima Peru; ^31^ Servicio de Cardiología Hospital Santo Tomas Ciudad de Panamá Panama; ^32^ Servicio de Cardiología Fundación Valle del Lili Cali Colombia

**Keywords:** Americas, epidemiology, heart failure, multicenter registry, Heart Failure, Epidemiology

## Abstract

**Background:**

Heart failure (HF) is a growing global health concern, particularly in low‐ and middle‐income countries. Contemporary, region‐specific data from the Americas remain scarce. The AMERICCAASS (Registro Americano de Insuficiencia Cardiaca Ambulatoria o Agudamente Descompensada [American Registry of Ambulatory or Acutely Decompensated Heart Failure]) registry was designed to provide broad, robust regional evidence on HF.

**Methods:**

The AMERICCAASS registry is a prospective, multicenter, observational study including adults with HF enrolled as inpatients or outpatients at 119 institutions in 21 countries across the Americas. Sociodemographic and clinical variables were summarized descriptively.

**Results:**

A total of 6732 patients were included; 30.1% were inpatients and 69.9% outpatients. The median age was 68.6 years, and 58.2% were men. Hypertension (69.6%), dyslipidemia (41.6%), and coronary artery disease (39.2%) were the most common comorbidities. Ischemic (39.9%) and hypertensive (17.9%) etiologies predominated, while Chagas disease was present in 3.4%. Unhealthy habits were common, with 39.8% reporting smoking, 24.4% alcohol use, and only 23.9% regular physical activity. New York Heart Association class III predominated in inpatients (45.2%), whereas New York Heart Association class II predominated in outpatients (52.6%). HF with reduced left ventricular ejection fraction was the most common phenotype in both settings: inpatients (56.8%) and outpatients (55.0%). β Blockers and mineralocorticoid receptor antagonists were the most frequently prescribed therapies, followed by sodium–glucose cotransporter‐2 inhibitors. Among inpatients, intensive care unit admission occurred in 55.9%, and the in‐hospital mortality rate was 7%.

**Conclusions:**

The AMERICCAASS registry offers a comprehensive overview of HF in the Americas. Although patient profiles were broadly consistent with global trends, important regional features were observed, including frequent unhealthy lifestyle habits and underuse of key therapies. The high prevalence of HF with reduced left ventricular ejection fraction, together with elevated intensive care unit admission rates and in‐hospital death, suggests potential gaps in care and underscores the need to further evaluate HF care delivery across the region.

**Registration:**

URL: clinicaltrials.gov; Unique Identifier: NCT05295641.

## Graphical abstract

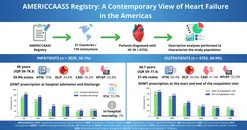

**Nonstandard Abbreviations and Acronyms**
AMERICCAASS: Registro Americano de Insuficiencia Cardiaca Ambulatoria o Agudamente Descompensada (American Registry of Ambulatory or Acutely Decompensated Heart Failure)
ARNI: angiotensin receptor–neprilysin inhibitor
BB: β blocker
G‐CHF: Global Congestive Heart Failure
GDMT: guideline‐directed medical therapy
HFpEF: heart failure with preserved ejection fraction
HFrEF: heart failure with reduced ejection fraction
INTER‐CHF: International Congestive Heart Failure
MRA: mineralocorticoid receptor antagonist
NYHA: New York Heart Association
REPORT‐HF: International Registry to Assess Medical Practice With Longitudinal Observation for Treatment of Heart Failure
SGLT2i: sodium–glucose cotransporter‐2 inhibitor

**Clinical Perspective**

**What Is New?**

- This is the first large‐scale, multinational registry to provide a contemporary regional overview of heart failure across the Americas, integrating both inpatient and outpatient populations from diverse health care settings.

**What Are the Clinical Implications?**

- Our findings reveal distinctive regional patterns, including a high burden of modifiable risk factors, underuse of guideline‐directed medical therapy, and substantial rates of intensive care unit admission and in‐hospital mortality among inpatients.
- These gaps identify concrete targets for prevention, optimization of evidence‐based therapies, and strengthening of heart failure care pathways and health systems tailored to the realities and inequalities of the Americas.

Heart failure (HF) is a significant health challenge worldwide due to its high prevalence, substantial morbidity and death, and the increasing burden it places on health care systems.^1^, ^2^ These challenges are further exacerbated by an increase in HF prevalence, especially among younger individuals.^3^ In low‐ and middle‐income countries, HF remains a leading cause of death and hospitalization, and its prognosis is often worse due to a combination of complex social determinants of health and limited access to optimal treatment.^4^ Latin American countries present a unique mix of HF risk factors, incorporating those commonly observed in developed countries along with additional challenges such as Chagas disease and rheumatic fever.^5^, ^6^

Despite this distinctive epidemiological profile and the fact that these countries bear ≈80% of the global cardiovascular disease burden, the majority of HF research continues to originate from the United States and Europe.^7^ Historically, cardiovascular research output has been concentrated in these regions, with more limited representation from Latin America.^8^ Consequently, much of the existing epidemiological and clinical data are either extrapolated from studies conducted in these high‐income settings or derived from small regional subsets within international registries, thereby limiting the accuracy of insights into the regional disease profile.^5^ Additionally, most available information from Latin America has been concentrated in Brazil and Argentina, with increasing contributions from Mexico and Colombia in recent years, while other countries in the region remain underrepresented.^5^, ^9^

AMERICCAASS (Registro Americano de Insuficiencia Cardiaca Ambulatoria o Agudamente Descompensada [American Registry of Ambulatory or Acutely Decompensated Heart Failure]) was established to address gaps in the epidemiological and clinical understanding of HF in the region. Its primary objective is to generate robust multicenter data that more accurately reflect the reality of HF in the Americas, thereby strengthening the characterization of sociodemographic patterns, risk factors, clinical progression, and patient outcomes across the continent. In this study, we present the final recruitment results of the registry, providing a comprehensive characterization of patients diagnosed with HF throughout the Americas. These findings provide essential data to inform evidence‐based public health policies and optimize therapeutic strategies tailored to regional needs.

## Methods

### Data Availability

The corresponding author will provide the raw data supporting this article's conclusions upon a reasonable request.

### Ethical Considerations

The study adhered to the principles outlined in the Declaration of Helsinki and received approval from the Comité de Ética en Investigación Biomédica (Biomedical Research Ethics Committee), the institutional Ethics Committee of Fundación Valle del Lili (Valle del Lili Foundation) (November 13, 2018/No. 313‐2018). The ethics committee of each participating institution approved the study protocol. As the coordinating institution, the Comité de Ética en Investigación Biomédica of Fundación Valle del Lili waived the requirement for patient consent, as no interventions were conducted on participants, and all data were collected from medical records. However, each participating institution had the discretion to implement informed consent as deemed appropriate.

### Study Design

The AMERICCAASS registry is a multicenter, observational, and prospective study involving health care institutions across several countries in the Americas, including North, Central, and South America and the Caribbean.^10^ Participating centers were health care institutions from countries affiliated with the Sociedad Interamericana de Cardiología (Inter‐American Society of Cardiology). Site participation was established through an open invitation disseminated via the Sociedad Interamericana de Cardiología network, and enrollment was voluntary. Thus, participating sites constituted a network‐based sample. The registry aims to characterize and evaluate the sociodemographic and clinical profiles of patients with HF in the region. HF diagnosis was established on the basis of documentation in medical records and subjected to the evaluation of participating principal investigators. Inclusion and exclusion criteria are summarized in Table 1.

**Table 1:** Inclusion and Exclusion Criteria for the AMERICCAASS Registry

Inclusion criteria
Patients aged ≥18 y Patients admitted to the inpatient setting with a diagnosis of acute HF Patients evaluated in the outpatient clinic with a diagnosis of HF

Exclusion criteria
Inpatients with HF not being the primary reason for inpatient admission Patients on a waiting list for a cardiac transplant Patients with long‐term ventricular assist devices or those undergoing implantation as destination therapy Patients in palliative care due to HF or another condition Patients discharged or transferred to another institution within 48 h of hospitalization Patients with a life expectancy of ≤6 mo due to a noncardiac condition

AMERICCAASS indicates Registro Americano de Insuficiencia Cardiaca Ambulatoria o Agudamente Descompensada (American Registry of Ambulatory or Acutely Decompensated Heart Failure); and HF, heart failure.

This registry was coordinated by the Board of Directors of the Consejo Interamericano de Falla Cardiaca e Hipertensión Pulmonar (Inter‐American Council on Heart Failure and Pulmonary Hypertension), a division of the Sociedad Interamericana de Cardiología, which served as the Scientific and Steering Committee. The Centro de Investigaciones Clínicas (Clinical Research Center) at Fundación Valle del Lili in Cali, Colombia, provided technical and logistical support for its implementation and development.

### Data Collection

Individuals aged ≥18 years with a confirmed HF diagnosis, either in the inpatient or outpatient setting, were included. No patient was included in >1 category. Recruitment began in April 2022 and concluded in November 2023. Sociodemographic and clinical data were gathered using electronic health records.

Information was collected for each participant from electronic medical records corresponding to outpatient visits or hospital admissions. Sociodemographic data, including self‐reported race, comorbidities, medical history, preadmission medication use, lifestyle habits, and the most recent left ventricular ejection fraction (LVEF) before evaluation, were recorded.

Lifestyle habits included smoking history, alcohol consumption, and physical activity. Smoking history was based on the presence of any past or current tobacco use as documented in the clinical history. Alcohol consumption was assessed on the basis of frequency. Physical activity was recorded as the number of days per week in which the patient reported performing any kind of exercise, irrespective of intensity or duration. The New York Heart Association (NYHA) functional class was assessed at hospital admission for inpatients and during the clinical visit for outpatients.

In inpatients, additional clinical information was collected, including the etiology of HF on the basis of prior history and diagnostic workup, the primary cause of the acute decompensation episode, the clinical presentation at admission, the hemodynamic profile according to clinical assessment, and the patient's condition at discharge. Pharmacological management at hospital discharge or therapeutic plans established during outpatient visits were also documented.

A principal investigator and a subinvestigator responsible for data collection represented each participating medical institution. Principal investigators were internists, cardiologists, or HF subspecialists, while subinvestigators were nursing staff, general practitioners, internists, cardiologists, or HF subspecialists. Participant information was stored on the Research Electronic Data Capture platform to ensure anonymity, confidentiality, and data security. Each participating institution was assigned a unique user name and password for secure access.

### Statistical Analysis

To comprehensively characterize the study population, descriptive statistical analyses were performed on sociodemographic and clinical variables. Continuous variables were summarized as medians with interquartile ranges to provide robust and clinically interpretable descriptive summaries. Categorical variables were presented as absolute frequencies and percentages.

Missingness was assessed across all variables, with most showing <1% missingness. Relevant missing data are reported, and variables were summarized using available observations. For analyses using LVEF‐based HF phenotypes, only patients with complete LVEF data were included. All statistical analyses were conducted using R version 4.1.1 (R Foundation for Statistical Computing, Vienna, Austria) and RStudio version 1.4.1717 (Posit PBC, Boston, MA).

For visual clarity, bar labels in figures were rounded to the nearest integer (≥ 0.5 rounded up) using standard rules, while exact values were provided in the main text to preserve analytical precision. Visualizations were created using BioRender.com, Python version 3.13.2 (Python Software Foundation, Wilmington, DE), and MapChart.net.

The registry's methodology, data management, statistical methods, and bioethical considerations have been detailed previously.^10^

## Results

Of 6798 patients in the original data set, 66 were excluded for data quality/eligibility reasons. Thus, 6732 patients were considered for the final analysis (Figure 1). These patients were enrolled at 119 medical institutions across 21 countries in the Americas (Figure 2). Detailed information on the number of participating centers and enrolled patients by country is provided in Table S1. Among these, 2029 patients (30.1%) were inpatients, and 4703 (69.9%) were outpatients. The median age was 68 (interquartile range, 58–78.3) years for inpatients and 68.7 (interquartile range, 59.0–77.4) years for outpatients. More than 30% of patients in the total cohort were aged ≥75 years, with a similar distribution for both settings. Sex distribution was similar across inpatient and outpatient settings, with men comprising the majority of the total population (58.2%). The most common ethnicity in both cohorts was Mestizo (Table 2).

**Figure 1:**
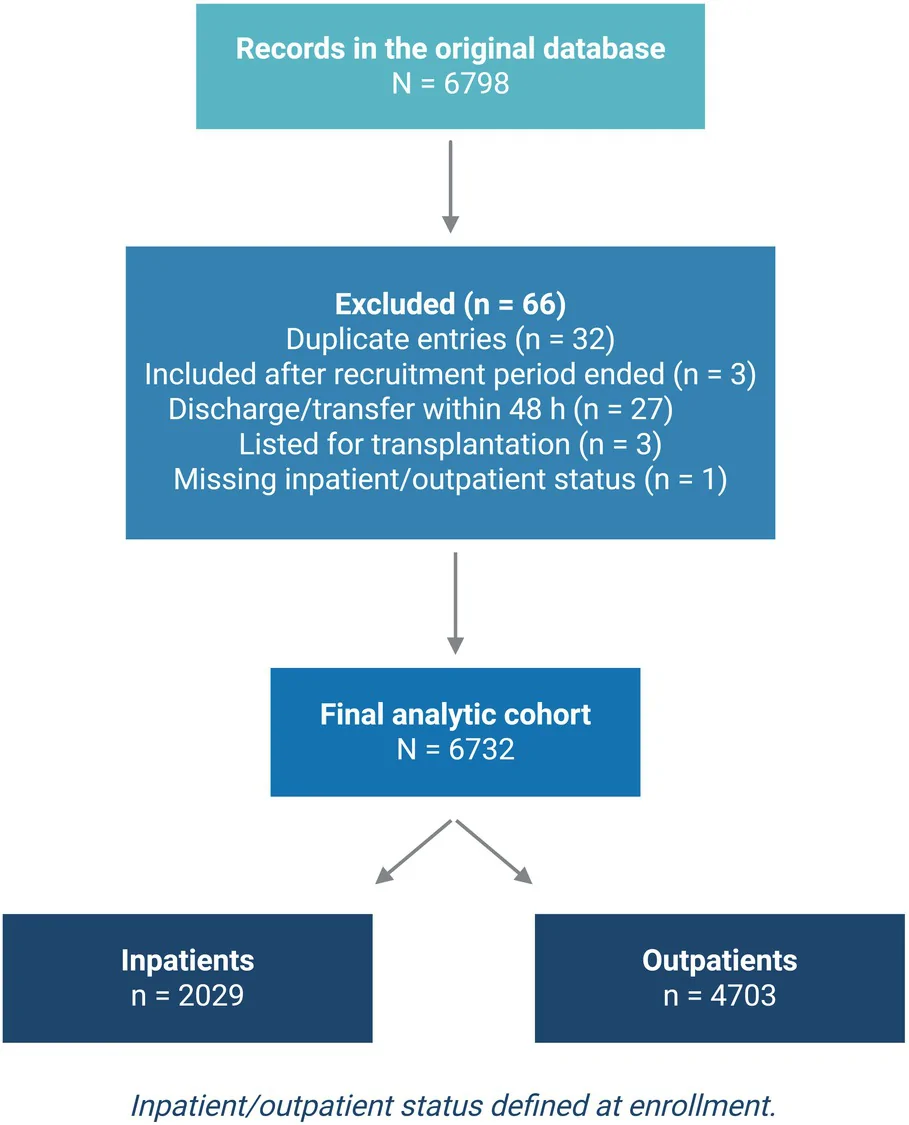
Study flow diagram and final analytic sample.

**Figure 2:**
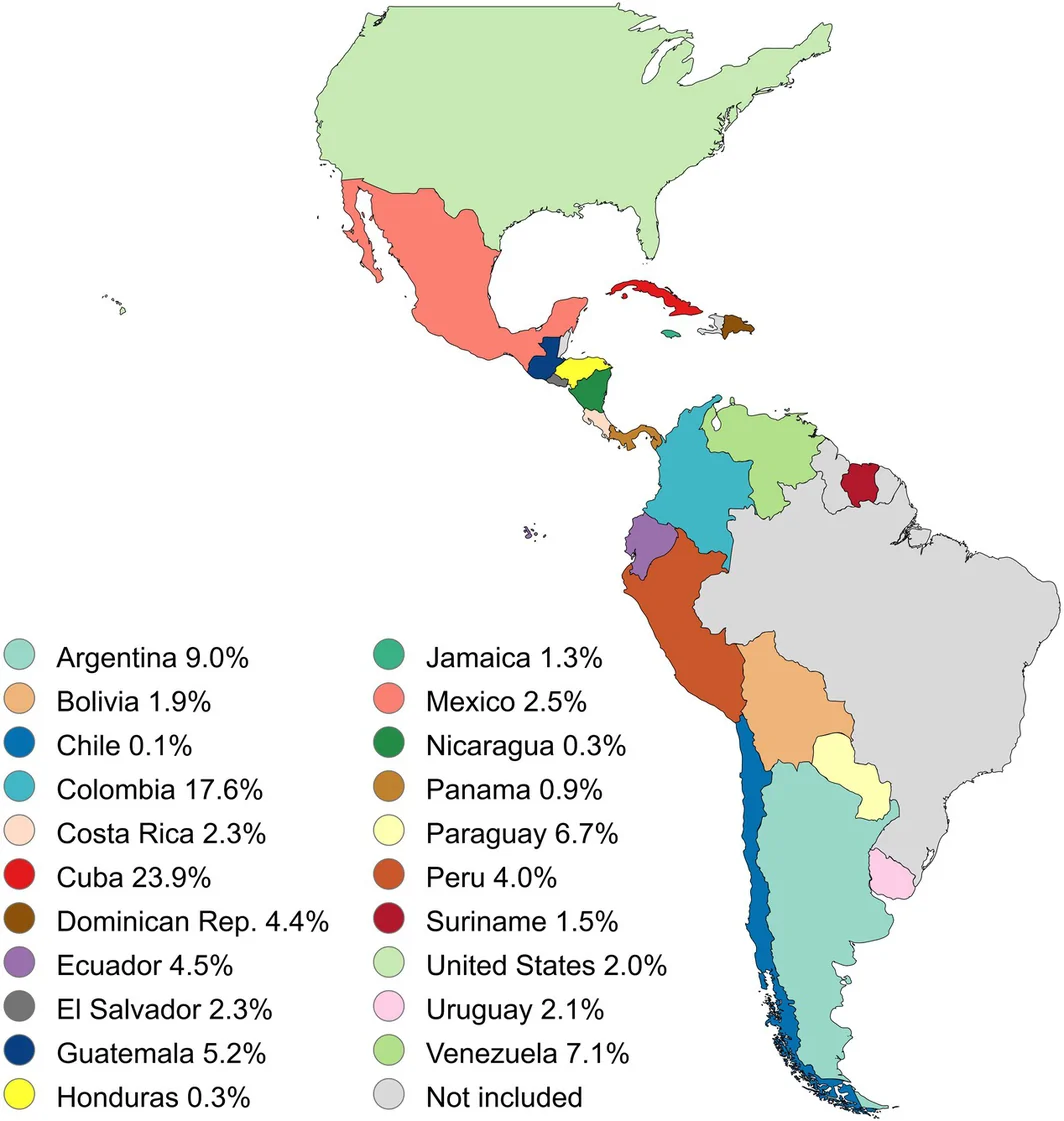
Countries with participating institutions and their recruitment rate. Not included: countries that did not contribute patient‐level data to the registry.

**Table 2:** Demographics and Baseline Comorbidities

Variable	Overall, N=6732	Inpatient, n=2029	Outpatient, n=4703
Age distribution*			
Age, y,* median (IQR)	68.6 (58.8–77.7)	68.0 (58.0–78.3)	68.7 (59.0–77.4)
Age category 18–54 y, n (%)	1185 (17.7)	388 (19.2)	797 (17.0)
Age category 55–64 y, n (%)	1523 (22.7)	476 (23.5)	1047 (22.4)
Age category 65–74 y, n (%)	1859 (27.7)	499 (24.6)	1360 (29.1)
Age category ≥75 y, n (%)	2134 (31.8)	662 (32.7)	1472 (31.5)
Sex distribution,* n (%)			
Men	3913 (58.2)	1216 (59.9)	2697 (57.4)
Women	2816 (41.8)	813 (40.1)	2003 (42.6)
Self‐reported race and ethnicity,* n (%)			
Mestizo	3543 (53.9)	1091 (54.6)	2452 (53.6)
White	2210 (33.6)	678 (33.9)	1532 (33.5)
Black	646 (9.8)	174 (8.7)	472 (10.3)
Native	110 (1.7)	40 (2.0)	70 (1.5)
Other	66 (1.0)	16 (0.8)	50 (1.1)
History of HF, n (%)			
New onset	2277 (33.8)	764 (37.7)	1513 (32.2)
Chronic	4455 (66.2)	1265 (62.3)	3190 (67.8)
Baseline medical history, n (%)			
Hypertension	4687 (69.6)	1421 (70.0)	3266 (69.4)
Dyslipidemia	2802 (41.6)	706 (34.8)	2096 (44.6)
Coronary artery disease	2640 (39.2)	694 (34.2)	1946 (41.4)
Diabetes	1901 (28.2)	624 (30.8)	1277 (27.2)
Atrial fibrillation	1742 (25.9)	552 (27.2)	1190 (25.3)
Severe valvular insufficiency	1183 (17.6)	358 (17.6)	825 (17.5)
Chronic kidney disease	1067 (15.8)	333 (16.4)	734 (15.6)
Cardiac device	810 (12.0)	186 (9.2)	624 (13.3)
Thyroid disorder	674 (10.0)	201 (9.9)	473 (10.1)
Peripheral artery disease	605 (9.0)	178 (8.8)	427 (9.1)
Chronic obstructive pulmonary disease	600 (8.9)	190 (9.4)	410 (8.7)
Severe valvular stenosis	353 (5.2)	120 (5.9)	233 (5.0)
Stroke/transient ischemic attack	530 (7.9)	149 (7.3)	381 (8.1)
OSAHS	306 (4.5)	60 (3.0)	246 (5.2)
Cancer	297 (4.4)	98 (4.8)	199 (4.2)
Lifestyle habits, n (%)			
Smoking	2681 (39.8)	818 (40.3)	1863 (39.6)
Previous	2255 (84.1)	660 (80.7)	1595 (85.6)
Current	426 (15.9)	158 (19.3)	268 (14.4)
Alcohol consumption	1642 (24.4)	530 (26.1)	1112 (23.6)
1–2 times/wk	606 (36.9)	207 (39.1)	399 (35.9)
3–5 times/wk	203 (12.4)	72 (13.6)	131 (11.8)
5–7 times/wk	93 (5.7)	41 (7.7)	52 (4.7)
Occasional social	740 (45.1)	210 (39.6)	530 (47.7)
Physical activity, n (%)	1606 (23.9)	314 (15.5)	1292 (27.5)
1–2 times/wk	739 (46.0)	162 (51.6)	577 (44.7)
3–5 times/wk	514 (32.0)	105 (33.4)	409 (31.7)
5–7 times/wk	353 (22.0)	47 (15.0)	306 (23.7)

HF indicates heart failure; IQR, interquartile range; and OSAHS, obstructive sleep apnea–hypopnea syndrome.

* Age was available in 6701 patients (99.5%); missing data: 31 overall (0.5%), including 27 outpatients and 4 inpatients. Sex was available in 6729 patients (99.9%); missing data: 3 overall (<0.1%), all in outpatients. Self‐reported race and ethnicity was available in 6575 patients (97.7%); missing data: 157 overall (2.3%), including 127 outpatients (2.7%) and 30 inpatients (1.5%).

The most prevalent comorbidities were hypertension (69.6%), dyslipidemia (41.6%), and coronary artery disease (39.2%). While hypertension prevalence was similar across settings, coronary artery disease and dyslipidemia were more common among outpatients than inpatients (41.4% versus 34.2% and 44.6% versus 34.8%, respectively). Diabetes and atrial fibrillation were also frequent. Overall, one third of patients had new‐onset HF, whereas two thirds had chronic HF. New‐onset HF was more frequent among inpatients, whereas chronic HF predominated among outpatients (Table 2).

Approximately 40% of the population reported a smoking history, with similar prevalence among inpatients and outpatients. Among them, the vast majority were former smokers. Alcohol consumption was reported by 24.4%, slightly more common among inpatients (26.1%) than outpatients (23.6%), with “occasional social use” being the most frequent pattern. Physical activity was reported by 23.9%, more frequently among outpatients (27.5%) than inpatients (15.5%) (Table 2).

The principal etiologies were ischemic (39.9%) and hypertensive (17.9%), with a similar distribution between both groups. However, the third most common cause differed by group, being valvular (12.1%) in inpatients and idiopathic (9.9%) in outpatients (Figure 3). Tachycardia‐induced cardiomyopathy (5.2%) and Chagas disease (3.4%) were also notable among the reported etiologies. Other etiologies accounted for a smaller proportion in both groups (Table S2).

**Figure 3:**
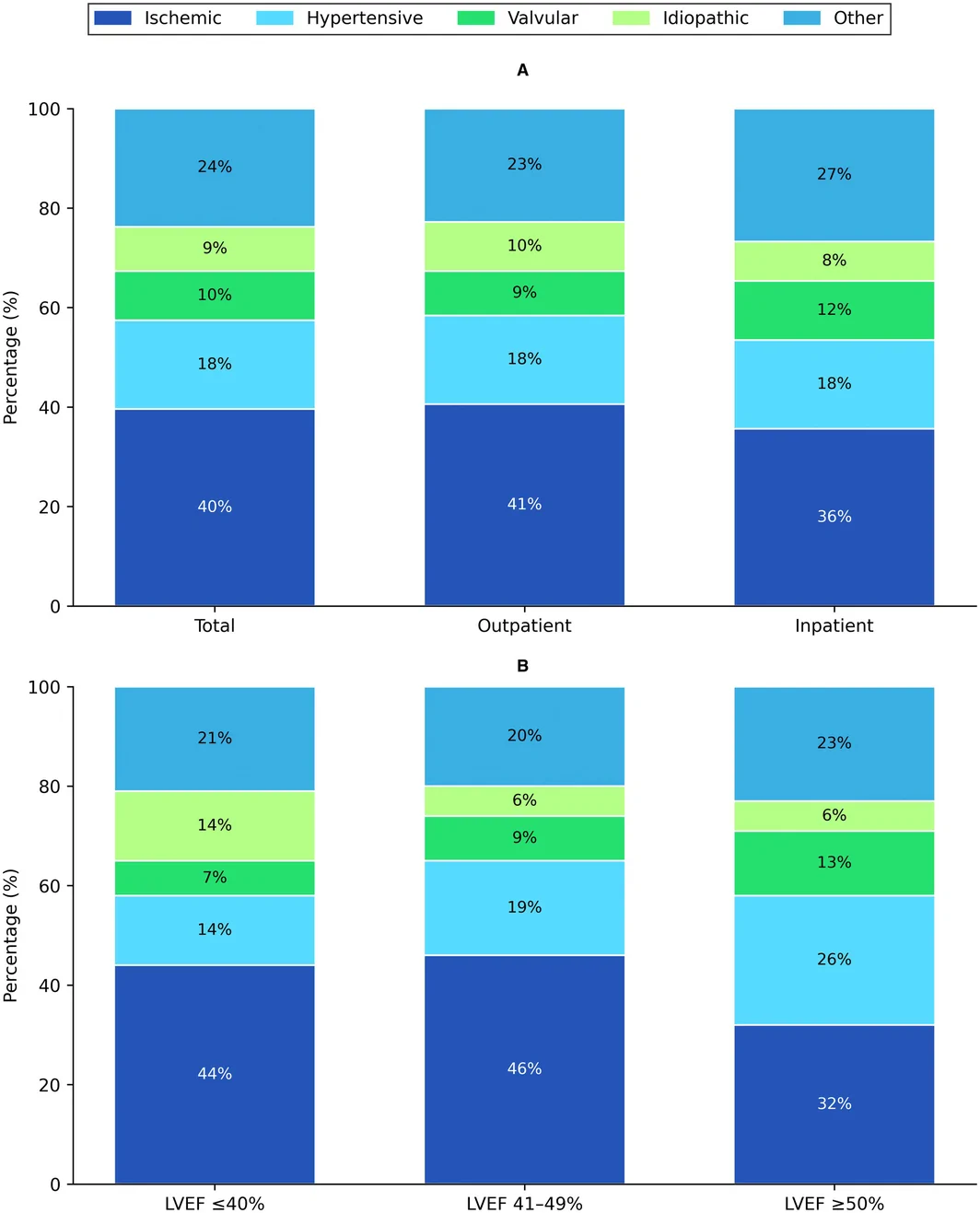
Main etiologies. **A**, Proportional distribution of HF etiologies in the total population and by care setting (outpatients vs inpatients). **B**, Proportional distribution of HF etiologies by LVEF category in outpatients only. “Other” includes less common causes such as alcohol, chemotherapy, viral infections, Chagas disease, hypertrophic and familial cardiomyopathies, and trauma, among others. HF indicates heart failure; and LVEF, left ventricular ejection fraction.

Among patients with available LVEF data, reduced LVEF was observed in 55.0% of outpatients, most of whom were classified as NYHA class II (52.6%) or III (24.0%). Among inpatients, reduced LVEF was present in 56.8%, with NYHA class III and IV comprising 45.2% and 30.1%, respectively. Importantly, LVEF data were missing in 26.0% of inpatients and 13.0% of outpatients. Disease progression was the leading cause of decompensation (38.2%), followed by acute coronary syndrome (13.5%), infections (9.6%), atrial fibrillation (9.0%), and poor pharmacological adherence (7.9%). Clinical presentation was predominantly acute decompensated HF (60.2%), with a warm/wet hemodynamic profile in 67.3%. Intensive care unit (ICU) admission occurred in 55.9% of cases, and the in‐hospital mortality rate reached 7% (Table 3).

**Table 3:** Clinical Characteristics

Variable	Inpatient, n=2029	Outpatient, n=4703
LVEF,* n (%)		
≤40	853 (56.8)	2252 (55.0)
41–49	215 (14.3)	719 (17.6)
≥50	433 (28.8)	1121 (27.4)
Unknown	528 (26.0)	611 (13.0)
NYHA class,* n (%)		
Class I	48 (2.4)	927 (20.7)
Class II	440 (22.3)	2356 (52.6)
Class III	892 (45.2)	1074 (24.0)
Class IV	593 (30.1)	122 (2.7)
Unknown	56 (2.8)	224 (4.8)
Cause of decompensation,* n (%)		
Disease progression	776 (38.2)	NA
Acute coronary syndrome	274 (13.5)	NA
Infection	194 (9.6)	NA
Atrial fibrillation	182 (9.0)	NA
Suboptimal adherence	160 (7.9)	NA
Other†	443 (21.8)	NA
Clinical presentation,* n (%)		
Acute decompensated HF‡	1176 (60.2)	NA
Pulmonary edema	281 (14.4)	NA
HF precipitated by acute coronary syndrome	184 (9.4)	NA
Cardiogenic shock	135 (6.9)	NA
Other, n (%)†	176 (9.0)	NA
Hemodynamic profile*		
Warm/wet, n (%)	1316 (67.3)	NA
Warm/dry, n (%)	300 (15.3)	NA
Cold/wet, n (%)	282 (14.4)	NA
Cold/dry, n (%)	58 (3.0)	NA
Hospital stay length, median (IQR)	9 (6–14)	NA
ICU admission,* n (%)		
Yes	1087 (55.9)	NA
No	857 (44.1)	NA
ICU stay length, median (IQR)	3 (1–6)	NA
Discharge condition,* n (%)		
Death	138 (7.0)	NA
Alive	1831 (93.0)	NA

HF indicates heart failure; ICU, intensive care unit; IQR, interquartile range; LVEF, left ventricular ejection fraction; NA, not applicable; and NYHA, New York Heart Association.

* Percentages for these variables were calculated on the basis of available data (excluding missing values). Missing data among inpatients included 77 (3.8%) for clinical presentation, 73 (3.6%) for hemodynamic profile, 85 (4.2%) for ICU admission, and 60 (3.0%) for discharge condition. Missing values for LVEF and NYHA class are shown in the table.

^†^ “Other” causes of decompensation include less frequent factors such as nonpharmacological nonadherence; ventricular arrhythmias; uncontrolled hypertension; renal dysfunction; bleeding; recent procedures; and various pulmonary, metabolic, or neurological conditions. For clinical presentation, it includes uncommon forms such as hypertensive, right‐sided, and high‐output HF.

^‡^ “Acute decompensated HF” refers to decompensation with congestion as the primary presentation.

Overall, outpatient visits showed increased prescribing of guideline‐directed therapies (GDMTs), including β blockers (BBs), mineralocorticoid receptor antagonists (MRAs), sodium–glucose cotransporter‐2 inhibitors (SGLT2is), and angiotensin receptor–neprilysin inhibitors (ARNIs). In contrast, angiotensin receptor blockers (ARBs) decreased modestly, while angiotensin‐converting enzyme inhibitor (ACEI) use remained stable (Figure 4A). Among inpatients, prescribing showed a similar pattern, increasing from admission to discharge, particularly for SGLT2is and MRAs, whereas ARB and ACEI use remained essentially unchanged (Figure 4B).

**Figure 4:**
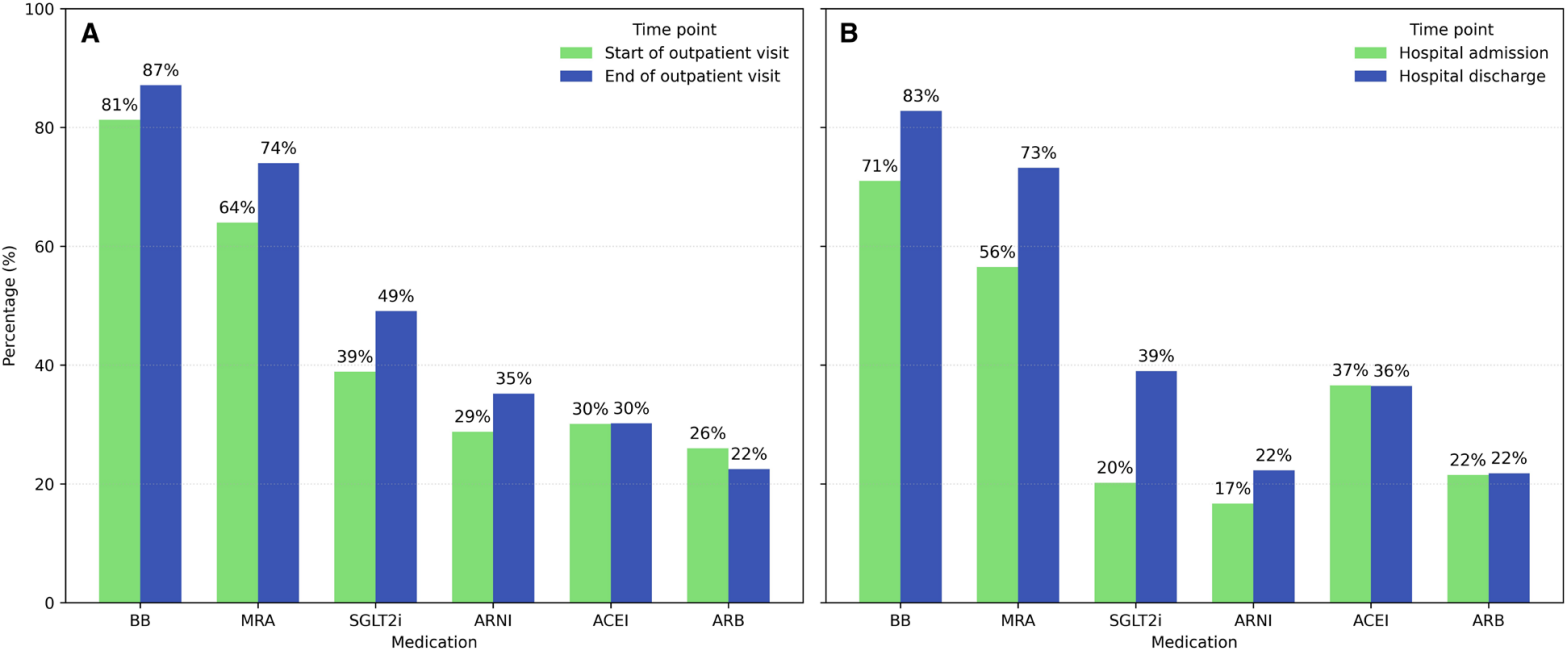
Changes in medication prescription in outpatient and inpatient settings. **A**, Changes in HF therapy prescription rates from the start to the end of outpatient visits, and (**B**) from hospital admission to discharge. In both (**A**) and (**B**), patients with de novo HF were excluded at baseline, and in (**B**), those who died before discharge were also excluded. Percentages were calculated on the basis of the total number of patients with available LVEF data at each time point. ACEI indicates angiotensin‐converting enzyme inhibitor; ARB, angiotensin receptor blocker; ARNI, angiotensin receptor–neprilysin inhibitor; BB, β blocker; LVEF, left ventricular ejection fraction; MRA, mineralocorticoid receptor antagonist; and SGLT2i, sodium–glucose cotransporter‐2 inhibitor.

When stratified by LVEF, outpatients with HF with reduced ejection fraction (HFrEF) showed increases in BB, MRA, SGLT2i, and ARNI use, stability in ACEIs, and a reduction in ARBs. In HF with mildly reduced ejection fraction, BB, MRA, and SGLT2i use also increased, with smaller gains in ARNIs and ACEIs, and a decline in ARBs. In HF with preserved ejection fraction (HFpEF), BB, MRA, and SGLT2i use rose, ARNI and ACEI use increased slightly, and ARB use decreased.

Among inpatients, HFrEF showed increases in BB, MRA, and SGLT2i use, moderate gains in ARNI use, and stable ACEI and ARB use. HF with mildly reduced ejection fraction exhibited a similar pattern, with the largest relative rise in SGLT2is. In HFpEF, BB, MRA, and SGLT2i use increased, ARNI use rose, and ACEI/ARB use remained stable. Detailed percentages for each HF group and LVEF category are provided in Figures 5 and 6.

**Figure 5:**
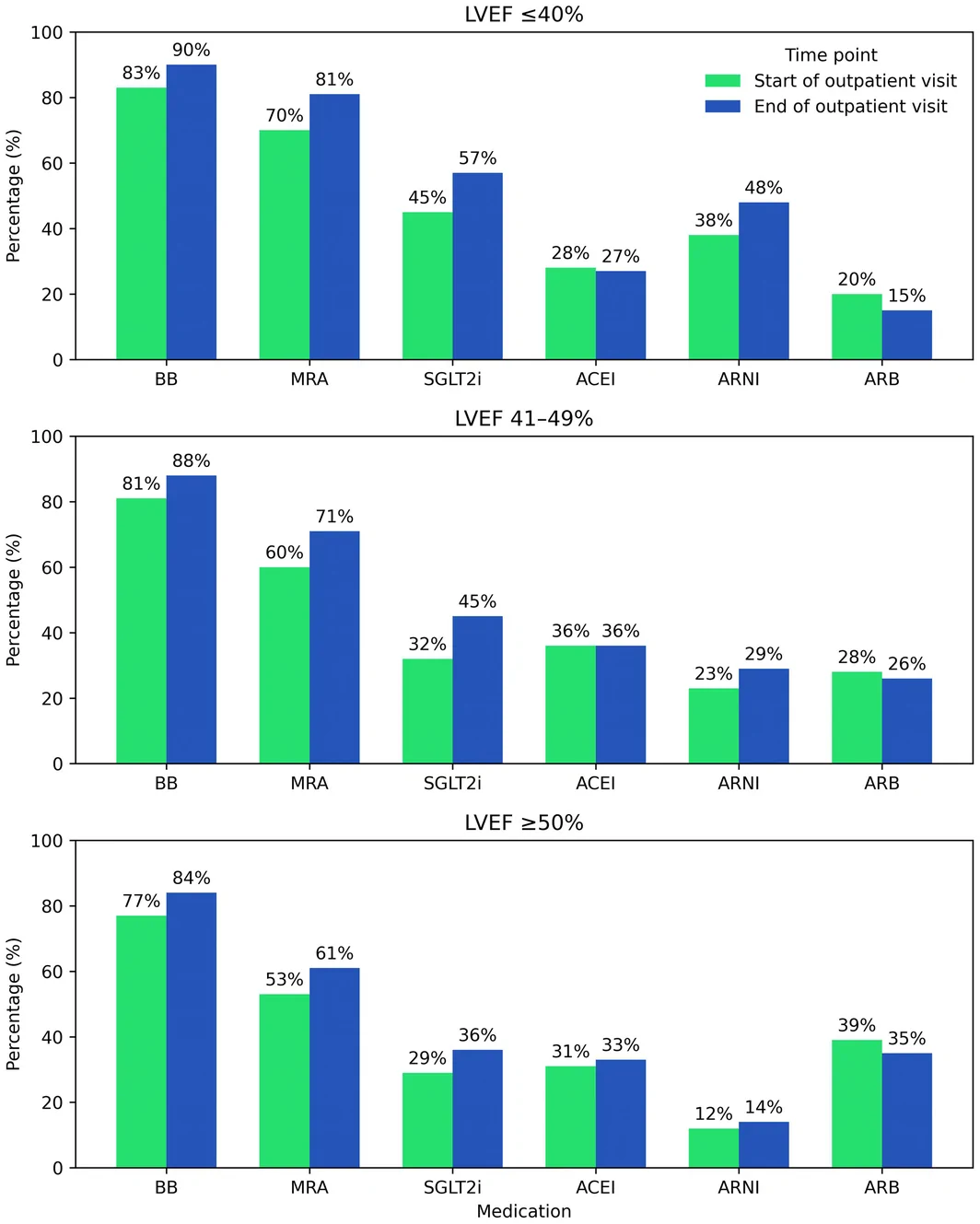
Changes in medication prescription in the outpatient setting, stratified by LVEF group. Patients with de novo HF were excluded from the analysis of medication prescription at the start of the visit. ACEI indicates angiotensin‐converting enzyme inhibitor; ARB, angiotensin receptor blocker; ARNI, angiotensin receptor–neprilysin inhibitor; BB, β blocker; LVEF, left ventricular ejection fraction; MRA, mineralocorticoid receptor antagonist; and SGLT2i, sodium–glucose cotransporter‐2 inhibitor.

**Figure 6:**
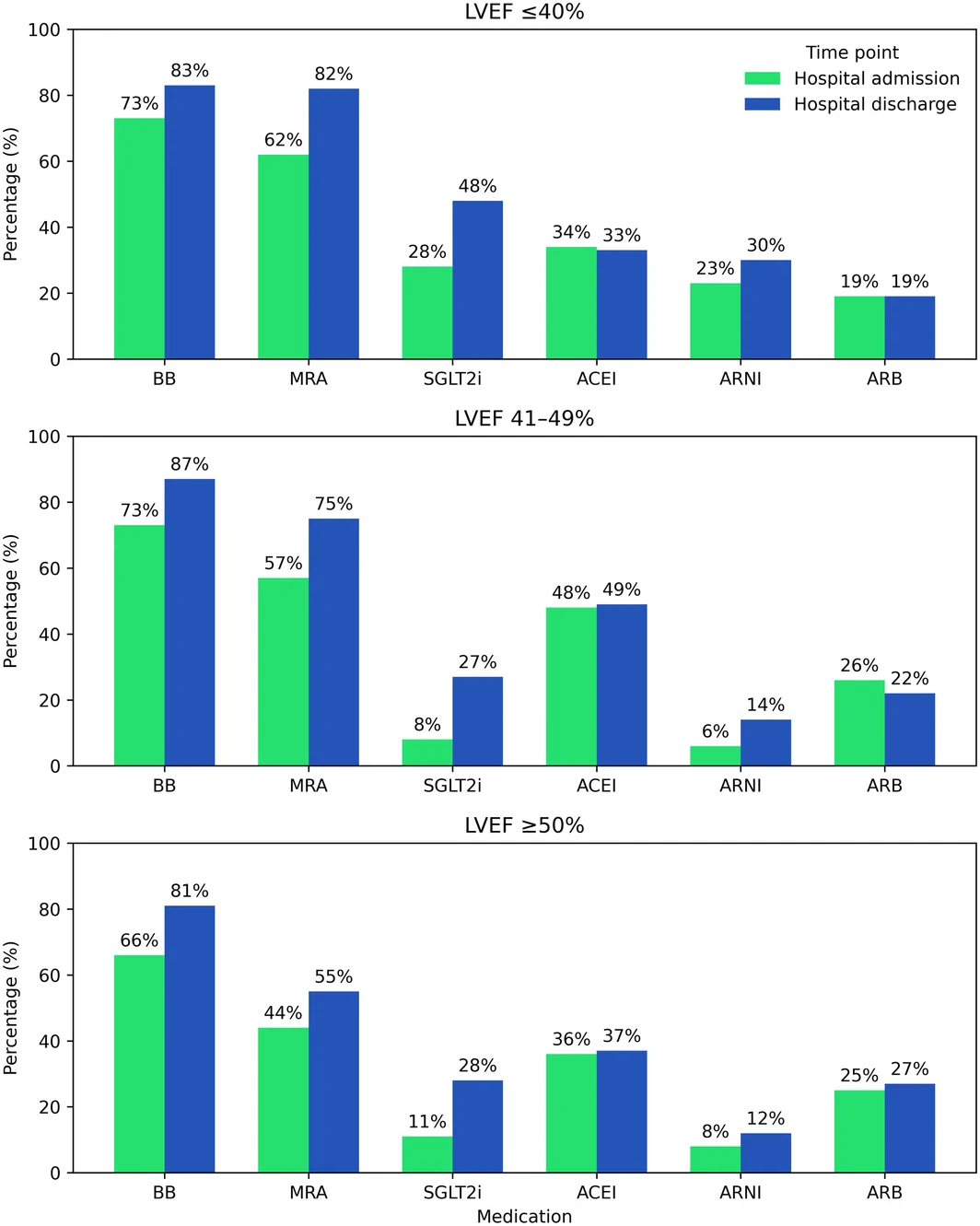
Changes in medication prescription in the inpatient setting, stratified by LVEF group. Medication use at hospital admission was calculated excluding patients with de novo HF, whereas patients who died during hospitalization were excluded from the discharge analysis. ACEI indicates angiotensin‐converting enzyme inhibitor; ARB, angiotensin receptor blocker; ARNI, angiotensin receptor–neprilysin inhibitor; BB, β blocker; LVEF, left ventricular ejection fraction; MRA, mineralocorticoid receptor antagonist; and SGLT2i, sodium–glucose cotransporter‐2 inhibitor.

## Discussion

The AMERICCAASS registry represents the first collaborative effort to characterize HF across multiple countries in the Americas. Our findings confirm that HF remains a major health challenge in the region, with several critical patterns emerging from the data. Notably, we observed high rates of unhealthy lifestyle habits, suboptimal use of GDMT, and a high prevalence of HFrEF. ICU admission rates were strikingly high, and in‐hospital death remained considerable. These findings highlight regional challenges and support context‐specific interventions to improve HF outcomes across the Americas.

The age distribution in our cohort aligns with evidence showing that HF is most prevalent among adults aged >60 years, who face a markedly higher risk than younger individuals.^11^ Several cohort studies have reported a mean age >60 years across regions, including the Americas, further supporting this association.^4^, ^12^, ^13^ National reports from Colombia, Argentina, and Brazil have also shown a similar age distribution.^14^, ^15^, ^16^ However, recent epidemiological trends suggest a shifting disease burden, with HF prevalence declining in adults aged ≥70 years but rising in those aged 20 to 69 years, notably increasing in the United States since 2016.^11^ Similar patterns of younger age at presentation have been observed in lower–middle‐income countries and across Latin America.^17^, ^18^ This trend may reflect the suboptimal management of cardiovascular risk factors from an early age, as well as the continued prevalence of neglected etiologies (eg, Chagas disease or rheumatic fever) and limited prevention strategies.^6^, ^19^, ^20^ However, its observation in recent European studies suggests a potential global trend, possibly linked to rising modifiable risk factors such as obesity and smoking in this age group.^21^, ^22^

The sex distribution observed in our study also aligns with global data, where men have been associated with a higher age‐standardized HF prevalence, as seen in several cohort studies.^4^, ^12^, ^13^, ^23^ Nevertheless, recent data show that women account for half of all cases worldwide.^24^ In fact, HF is more prevalent in women aged >70 to 80 years than in men of the same age.^11^, ^23^, ^24^, ^25^, ^26^ This higher prevalence in women is likely explained by their longer life expectancy and a greater burden of specific risk factors, such as obesity.^25^ In our population, women comprised 41.8% of the cohort. While this may suggest an emerging trend, the higher male prevalence could reflect the distinctive epidemiology of HF in Latin America, where the condition tends to occur at younger ages and men are more commonly affected.^18^ For instance, the previously cited national reports also describe a male predominance.^14^, ^15^, ^16^

Well‐established HF risk factors, such as hypertension, dyslipidemia, coronary artery disease, and diabetes, were highly prevalent in both inpatient and outpatient settings.^25^ These findings remain clinically significant, as comorbidities such as coronary artery disease and diabetes are strongly associated with increased death, higher readmission rates, and poorer quality of life and self‐care in patients with HF.^27^ The G‐CHF (Global Congestive Heart Failure) registry found a similar distribution for these conditions on a global scale, independent of the clinical setting.^13^ Other large‐scale studies have identified these conditions as prevalent in the Americas and beyond.^4^, ^12^ However, subtle differences in comorbidity patterns across populations have been described and highlight the potential geographic heterogeneity of HF. For instance, Latin America appears to have a lower comorbidity burden than regions like Western Europe, likely due to earlier HF onset and differing etiologies.^28^ Supporting this, recent studies on comorbidity clustering have observed a younger and less comorbid cluster predominating in Latin America.^29^

Unhealthy lifestyles were highly prevalent in our cohort, a key concern given their association with HF risk and adverse outcomes. A study in patients without major cardiovascular disease found that unhealthy habits independently contributed to incident HF, regardless of metabolic or genetic risk.^30^ A study of 989 080 hospitalized patients with HF found that 15% had substance use disorders (12.1% tobacco related, 3.5% alcohol related). Notably, most were aged 45 to 55 years, reflecting age‐related epidemiological shifts.^31^ Importantly, these behaviors are associated with higher cardiovascular risk and death.^32^ Although these habits are common globally, the INTER‐CHF (International Congestive Heart Failure) study reported the highest rates among South American patients, highlighting a concerning regional burden that may worsen HF outcomes and reinforcing the relevance of our findings.^12^, ^13^ Given their well‐established impact on HF progression, improving targeted preventive strategies is crucial to mitigating their effects.

Hypertensive and ischemic etiologies, both well‐established contributors to incident HF, were highly prevalent.^33^ Large cohort studies further support these findings, identifying these pathogeneses as leading global causes of HF.^4^, ^12^, ^13^ However, regional differences in their burden may highlight geographic variations in risk factor distribution. For instance, hypertension has been identified as the leading cause of HF in the Americas, although this was not observed in our study.^34^ Chagas disease is a major cause of HF in Central and South America, representing a significant public health concern in Latin America.^35^ Although not negligible, the 3.4% prevalence of Chagasic cardiomyopathy in our cohort contrasts with reports identifying Chagas disease as a major HF contributor in the Americas.^35^, ^36^ This lower prevalence may be explained by the absence of Brazil in our registry, a country with one of the highest burdens of the disease.^36^ The urban location of most centers, along with underdiagnosis or limited screening, may also explain this finding. Nonetheless, Chagas disease remains a major regional cause of HF due to its association with poorer outcomes.^37^ Similarly, valvular heart disease, particularly from rheumatic fever, is a distinctive cause of HF in the Americas and may explain the 9.6% of cases in our cohort.^17^, ^38^ Finally, limited access to advanced imaging techniques may hinder the diagnosis of inflammatory or infiltrative cardiomyopathies, leading to more idiopathic cases.

Reduced LVEF was common among patients with available LVEF data in both care settings, underscoring HF severity in our cohort. Similarly, the G‐CHF registry, which included a similar proportion of hospitalized and ambulatory patients, also identified HFrEF as the most prevalent HF subtype in both groups.^13^ The REPORT‐HF (International Registry to Assess Medical Practice With Longitudinal Observation for Treatment of Heart Failure) study found that half of its population had HFrEF, with a consistent distribution across regions, except for Western Europe, where the prevalence was lower.^4^ The INTER‐CHF study also reported that 56.9% of its population had reduced LVEF; however, regional differences were observed, with a higher prevalence in Asia and other regions compared with South America. Notably, they defined reduced LVEF as <50%, which may partially explain these variations.^12^ The high prevalence of HFrEF in our region is particularly concerning, as this subtype has been associated with worse outcomes.^4^, ^39^

Given the nonnegligible proportion of missing LVEF data, HF phenotype estimates should be interpreted with caution, as they reflect only patients with available measurements. As shown in Table S3, LVEF missingness was uneven across subregions and associated with differences in HF onset status, comorbidity burden, and NYHA class completeness. These findings suggest that LVEF data were not collected or documented uniformly across the cohort and may reflect variability in echocardiographic availability and documentation practices across centers. In some resource‐limited settings, this may also have led to more selective prioritization of echocardiographic assessment in patients with more established disease or greater clinical complexity.

In our study, NYHA classes II and III were the most common classifications, a pattern also observed in both global and regionally stratified data.^4^, ^12^, ^13^ As expected, inpatients more frequently presented with advanced NYHA classes compared with outpatients. The G‐CHF study reported similar patterns, with NYHA class III and IV more common in hospitalized patients.^13^ However, we observed nearly twice the proportion of NYHA class IV among inpatients, indicating greater symptom severity and functional impairment. This may reflect trends in lower–middle‐income countries, where patients often present with more advanced HF symptoms.^17^ It may also reflect more severe clinical conditions and be related to the high ICU admission rates and in‐hospital mortality rates reported in South America.^18^ In fact, the in‐hospital mortality rate observed in our study exceeds that reported in studies from other regions.^40^, ^41^ Moreover, hospitalized patients in lower–middle‐income countries face a 3‐ to 5‐fold higher short‐term mortality risk, likely reflecting care deficiencies that adversely affect outcomes in the region.^17^

Our findings indicate low use of GDMT such as SGLT2is, ACEIs, ARBs, and ARNIs, in contrast with the high use of BBs and MRAs in both settings. The greater BB use has also been observed in middle‐income countries.^17^ A similar pattern was observed in the INTER‐CHF study among South American patients, where ACEIs and ARBs were less frequently used than BBs and MRAs. However, their usage rates were higher than in our study, particularly for ARBs. In other regions, ARBs were also underused, showing patterns similar to ours. Interestingly, ACEI usage in those regions was comparable with MRA use.^12^ The CHAMP‐HF registry, which included outpatients with HFrEF, found that 67% of eligible patients received BBs, 33.4% were treated with MRAs, 60.5% received ACEIs/ARBs, and 13% were treated with ARNIs, highly comparable with our findings.^42^ The G‐CHF study showed a similar pattern for recommended therapies.^13^ Various studies have reported underuse of SGLT2is in clinical practice, a pattern also reflected in our findings.^43^, ^44^ This may partly reflect their recent inclusion in clinical guidelines and limited availability in some health care systems.^45^ This is particularly concerning given that these therapies have the strongest evidence for HFrEF, the most common HF subtype in our cohort.

When patients with available LVEF data were stratified by HF phenotype across both clinical settings, distinct patterns in therapy uptake emerged. In HFrEF, the use of BBs, MRAs, ARNIs, and SGLT2is was higher, partially aligning with guideline recommendations, although overall use remained relatively low. In HF with mildly reduced ejection fraction, the use of these medications was lower, except for ACEIs and ARBs, which were used more frequently than ARNIs. This pattern may reflect physician uncertainty about the management of this HF phenotype. As anticipated, patients with HFpEF received fewer renin–angiotensin–aldosterone system inhibitors, MRAs, and BBs, consistent with weaker guideline recommendations. Interestingly, SGLT2i use was low at baseline despite being the only therapy strongly supported by guidelines, which may reflect limited physician familiarity with the complex and evolving management of HFpEF.

Our results showed that both hospitalization and outpatient visits led to greater use of GDMT, particularly SGLT2is, MRAs, and ARNIs. Hospital admission offers a key opportunity to initiate or adjust evidence‐based medications. Notably, prescription rates at discharge in our inpatient cohort were comparable with those reported in middle‐income countries in the REPORT‐HF study, especially for BBs and MRAs.^4^ Outpatient visits similarly supported therapy initiation, titration, and adherence monitoring. The observed trends in ACEI and ARB prescription may reflect an evolving therapeutic strategy, in which ARNIs are increasingly preferred over traditional renin–angiotensin–aldosterone system inhibitors.^1^, ^2^ Importantly, the higher prescription rates of core quadruple therapy components in our HFrEF and HF with mildly reduced ejection fraction cohorts align with current guidelines and may differ from recent data from high‐income countries, where <10% of patients are discharged on all 4 classes.^46^ This suggests a positive trend toward broader therapeutic implementation and underscores the roles of both care settings in advancing evidence‐based HF management. In HFpEF, the increased use of SGLT2is at discharge and postvisit is consistent with guideline recommendations. Likewise, the higher prescription of MRAs and ARNIs may relate to emerging evidence of benefit in patients with LVEF <60%, although our results predate these reports.^47^, ^48^, ^49^

Despite this positive impact, the implementation of GDMT remains suboptimal. Clinician‐ and health system–level barriers may contribute to this gap, including limited familiarity with evolving recommendations, competing clinical demands, therapeutic inertia, limited capacity for timely treatment optimization, and insurance‐related constraints.^50^, ^51^ Additionally, the rapid evolution of medical knowledge may outpace health care systems' ability to integrate new therapies.^52^ However, INTER‐CHF data suggest that these factors alone may not fully explain the gap, as South American patients had the lowest rates of being uninsured and lacking medication coverage, pointing to other region‐specific influences that warrant further evaluation.^12^ Patient‐level barriers may also contribute to suboptimal GDMT uptake, such as limited understanding of the role and benefits of therapy, concerns about its safety, and high out‐of‐pocket costs, particularly in resource‐limited settings across the Americas.^12^, ^25^, ^50^, ^51^ Finally, sociodemographic disparities may also influence treatment implementation, as prior studies have reported lower use of ACEIs or ARBs in women and reduced ARNI use among Hispanic patients.^42^, ^53^

Our findings offer valuable insights into the current state of HF in the Americas. The younger age at presentation suggests a greater cumulative disease burden, likely reflecting early onset of cardiovascular risk factors and underscoring the need for earlier prevention. The high prevalence of unhealthy lifestyle habits highlights opportunities for nonpharmacological interventions. The elevated in‐hospital mortality rate and ICU admission rates, together with the high prevalence of reduced LVEF and advanced NYHA class, reflect the clinical severity of HF in the region and point to the need for improved acute management. Despite opportunities to initiate and optimize evidence‐based therapies, GDMT remains underused. The persistence of neglected etiologies, such as Chagas disease, highlights the region's unique epidemiological challenges and the need for focused research. These findings call for urgent, tailored strategies to improve prevention, treatment adherence, and health care equity across the Americas. Possible differences across subregions, as presented in Table S4, underscore the need for more granular geographic analyses to better characterize HF presentation, management, and outcomes across the Americas.

## Strengths and Limitations

Our study reinforces existing evidence on HF, particularly in the Americas, where data remain limited. By characterizing 6732 patients, this research offers a comprehensive and multinational perspective on HF across the Americas. The multicenter and prospective design enables the collection of standardized and up‐to‐date data, minimizing retrospective bias and enhancing the quality of information on the sociodemographic and clinical profiles of these patients. Furthermore, this approach strengthens the robustness and scope of our findings.

Nonetheless, the study has limitations. As a descriptive analysis, the study cannot establish causality or explore factors underlying the observed findings. Regions with limited resources and restricted health care access may be underrepresented, potentially biasing the data set toward better‐resourced settings and affecting the generalizability of the findings. Although the AMERICCAASS registry provides broad geographic coverage through the Sociedad Interamericana de Cardiología network, participation followed an open invitation and voluntary enrollment; therefore, it represents a network‐based sample that may not be nationally representative within each participating country nor fully representative of the broader Americas region. Notably, the absence of Brazil, a key contributor to cardiovascular research in the Americas, may limit the applicability of our findings, particularly for conditions such as Chagas disease. Relying on HF diagnoses based on principal investigator judgment could introduce variability and potentially affect our results; however, this risk is mitigated by the fact that all were internists, cardiologists, or HF subspecialists. Furthermore, some variables rely on self‐reported data, introducing a potential risk of recall and social desirability bias. Finally, as the data were extracted from medical records, variations in documentation practices and potential missing or incomplete information may have influenced the results.

## Conclusions

To our knowledge, this is the first study to comprehensively characterize patients with HF across the Americas, highlighting the value of international initiatives such as the AMERICCAASS registry. While sociodemographic and clinical profiles broadly align with global data, regional trends warrant specific attention. Unhealthy lifestyle habits are prevalent, posing major challenges for prevention and management. Despite opportunities to optimize GDMT during hospitalization and outpatient visits, key treatments such as SGLT2is, ACEIs, ARBs, and ARNIs remain underused. The high prevalence of reduced LVEF, together with the elevated ICU admission rates and in‐hospital mortality rates emphasizes the urgent need for better care. Addressing these gaps through targeted interventions, improved medication optimization, and risk factor control is essential to enhance HF outcomes in the region.

## APPENDIX

**AMERICCAASS Research Group Investigators**

The following individuals are members of the AMERICCAASS Research Group and include the Registry Principal Investigator, members of the Scientific Committee, and principal investigators from participating centers.

**Scientific Committee:** Juan Esteban Gómez‐Mesa, Fundación Valle del Lili (Principal Investigator of the AMERICCAASS Registry); Eduardo R. Perna, Instituto de Cardiología J. F. Cabral; Víctor Rossel, Hospital del Salvador/Instituto Nacional del Tórax; Daniel Quesada‐Chaves, Hospital San Vicente de Paul; Mario Speranza, Hospital Clínica Bíblica; Mark H. Drazner, University of Texas Southwestern Medical Center; Walter Alarco, Servicio de Cardiología, Instituto Nacional Cardiovascular INCOR, Lima, Perú; Alexander Romero‐Guerra, Hospital Santo Tomás; Sebastián Seni‐Molina, Fundación Valle del Lili (General Coordinator).

**Principal investigators from participating centers**

**Argentina:** Beder Gustavo Farez, Clínica Cerhu, Hospital Central Ramón Carrillo; Cristian Núñez‐Carrizo, Clínica El Castaño—CIMAC; Norberto Raúl Bornancini, Sanatorio Santa Clara Talar; Elda Lorena Brocal Ocampo, Establecimiento Asistencial Gobernador Centeno; Leandro Parrilla, Hospital General de Agudos Carlos G. Durand; Daniela García Brasca, Hospital Italiano de Córdoba; Juan Pablo Allende, Hospital Público Descentralizado Dr Guillermo Rawson; Ana Virginia Figueroa, Hospital Privado Universitario de Córdoba; Luis Guillermo Muñoz, Hospital San Bernardo; Juan Pablo Escalante, Instituto Cardiovascular de Rosario; Eduardo R. Perna, Instituto de Cardiología de Corrientes J. F. Cabral; Domingo César Azcona, Hospital Público Descentralizado Dr Marcial Vicente Quiroga; Alberto A. Fernández, Sanatorio Modelo Quilmes; Adrián Lescano, Sanatorio Trinidad Quilmes y Centro Gallego; Luis Roberto Lema, Instituto Modelo de Cardiología Privado SRL; José René Romano, Clínica Vrsalovic—Clínica del Angelo.

**Bolivia:** Orlando David Fernández‐Flores, Caja Petrolera de Salud; Juan Justiniano Encina, Hospital Universitario Japonés.

**Chile:** Víctor Rossel, Instituto Nacional del Tórax.

**Colombia:** Julián Lugo‐Peña, Clínica del Occidente; Gustavo Andrés Ortega Ramírez, Clínica General del Norte; Erika María Martínez Carreño, Clínica Iberoamérica; Alex Rivera‐Toquica, Clínica Los Rosales; Sebastián Campbell‐Quintero, Clínica Medilaser; Álvaro Mauricio Quintero‐Ossa, Clínica Cardio VID; Luis E. Echeverría, Fundación Cardiovascular de Colombia; María Juliana Rodríguez‐González, Fundación Cardioinfantil; Andrés Felipe Buitrago Sandoval, Hospital Fundación Santa Fe; Juan Esteban Gómez‐Mesa, Fundación Valle del Lili; Guillermo Trout‐Guardiola, Instituto Grupsalud; Gustavo Adolfo Moreno Silgado, Hospicardio; Carlos Hernán Calderón Franco, Hospital Santa Clara; Erwin Eladio Silva Cardona, Sociedad Cardiológica Colombiana S.A.S; Germán Camilo Giraldo‐González, Instituto Todo por el Corazón; Juan Alberto Cerón Benavides, Instituto Unimedic IPS; Alexis José Bastardo López, Clínica Avidantis S.A.S.

**Costa Rica:** Esteban Zavaleta Monestel, Hospital Clínica Bíblica; Andrés Ulate Retana, Hospital México CCSS; Daniel Quesada‐Chaves, Hospital San Vicente de Paul.

**Cuba:** Aymé Alberna Cardoso, Hospital Provincial Antonio Luaces Iraola; Tessa Negrín Valdés, Hospital Provincial Camilo Cienfuegos; Migdalia Cristina Cóbas Pérez, Hospital Provincial Clínico Quirúrgico Docente Faustino Pérez; Maité Goberna Rocha, Hospital Clínico Quirúrgico Docente Calixto García Íñiguez; José Carlos Acosta Abad, Hospital Provincial Docente Martin Chang Puga; Sucel Lázara Figueredo Carrazana, Hospital Provincial Clínico Quirúrgico Docente Carlos Manuel de Céspedes; José Carlos López Martín, Cardiocentro Santiago de Cuba; Mailyn Vilaú Jiménez, Hospital Provincial Clínico Quirúrgico Docente Abel Santamaría; Annia María Carrero‐Vásquez, Hospital Clínico Quirúrgico Docente Dr Enrique Cabrera; Maikel Santos Medina, Hospital General Docente Dr Ernesto Guevara de la Serna; Sandra Companioni Montero, Hospital Clínico Quirúrgico Docente Héroes del Baire; Yudmila Borges, Instituto Cardiología y Cirugía Cardiovascular; Elizabeth Sellén Sanchén, Hospital Provincial Clínico Quirúrgico Docente Manuel Ascunce Domenech; Tahimí Chacón Quesada, Hospital General Orlando Pantoja Tamayo; Yilian Herrera Domínguez, Hospital Provincial Saturnino Lora; Elién García Sánchez, Hospital Clínico Quirúrgico Docente Iván Portuondo; Raciel Victores Chacón, Hospital Provincial Clínico Quirúrgico Docente León Cuervo Rubio.

**Ecuador:** Harold Segundo Cedeño Macías, Hospital Centro Cardiológico A.C.I.; Fausto Bernardo Heredia Villacreses, Centro de Diagnóstico y Tratamiento de Enfermedades Cardiovasculares Cardioheredia; Manuel Hipólito Chaple La Hoz, Centro Médico Especialidades AMEDS; Jorge Gustavo Castro Daul, Clínica Jesús de Nazareth/Hospital San Marcos; Ricardo Mármol Alvear, Hospital Clínica Kennedy; David Alejandro Puga Bermúdez, Hospital José Carrasco Arteaga, Instituto Ecuatoriano de Seguridad Social; Marlon Patricio Aguirre Espinosa, Hospital Metropolitano; Nadia Sánchez, Hospital Hospicor; Leonardo Bermúdez, Hospital General IESS Portoviejo; Freddy Pow‐Chon‐Long, Hospital Luis Vernaza.

**El Salvador:** María Eugenia Menjívar‐De Ramos, Clínica Cardiología y Ortopedia; Juan Carlos Henríquez Bonilla, Clínica del Corazón Sinusal; Silvia Carolina Lazo‐Majano, Instituto Salvadoreño del Seguro Social.

**United States:** Lauren Cooper, North Shore University Hospital, Northwell Health; Elizabeth Ashley Hardin, University of Texas Southwestern Medical Center.

**Guatemala:** Milton Lubeck Herrera, Clínica HART; Luis Arturo Rodríguez, Hospital Roosevelt; Mádelyn Raquel Valle‐Ramos, Hospital Regional de Zacapa; Pablo José González Velásquez, Hospital San Juan de Dios; Sergio Roberto Fausto Ovando, Cardiocentro Chimaltenango.

**Honduras:** Emilio Samael Peralta‐López, Cardio Medical Center; Emilio Samael Peralta‐López, Instituto Nacional Cardiopulmonar.

**Jamaica:** Felix Nunura Arrese, Heart Institute of the Caribbean.

**México:** Eduardo Heberto Herrera Garza, Hospital Christus Muguerza Alta Especialidad; Jesús Eduardo Sánchez Hernández; José Benito Álvarez Mosquera, Hospital Español; Eufracino Sandoval Rodríguez, Hospital Miguel Hidalgo; Amada Álvarez‐Sangabriel, Instituto Nacional de Cardiología Ignacio Chávez; Clemente Barron Magdaleno, Instituto Nacional de Ciencias Médicas y Nutrición; Luis Carlos Pérez Plata, TecSalud Escuela de Medicina y Ciencias de la Salud del ITESM; Nilda Espinola‐Zavaleta, Servicios Profesionales del Sagrado Corazón de Jesús S.C.

**Nicaragua:** Pablo Hurtado Nuñez, Hospital Carlos R Huembes, Clínica Plaza España y Clínica Centro Médico Juigalpa.

**Panamá:** Gabriel Frago, Hospital Santo Tomás.

**Paraguay:** Claudine Judith Coronel Mariño, Clinicor Estudios Cardiológicos; Guillermo Oviedo‐Pereira, Hospital Central IPS; Javier Galeano Figueredo, Hospital de Clínicas Facultad de Ciencias Médicas; Luz Teresa Cabral‐Gueyraud, Hospital Nacional de Itauguá; Miguel A. Quintana, Instituto Cardiovascular del Sanatorio Migone; Ida Fabiola Rodríguez‐Caballero, Hospital Nacional de Cardiología “Profesor Doctor Juan Adolfo Cattoni”; Sergio Gabriel Vergara, Sanatorio Adventista de Asunción; María Paniagua, Sanatorio Italiano.

**Perú:** Miguel Eduardo Azañedo Velásquez, Instituto Cardiovascular Clinicor Norte/Hospital Nacional Sergio E. Bernales; Guillermo Alberto Arana Morales, Hospital III Chimbote EsSalud; Carlos Demetrio Renato Pacheco Bancayan, Luis Heysen Inchaustegui—EsSalud; Luis Felipe Anhuaman‐Atoche, Hospital María Auxiliadora; Paola Gissela Oliver Rengifo, Hospital Nacional Arzobispo Loayza; Juan Carlos More Terán, Hospital Nacional Daniel Alcides Carrion.

**Dominican Republic:** Ruddy Miguel García‐Safadit, Centro Médico Siglo 21; Sarah Raquel Marte‐Arias, Clínica Universitaria Unión Médica del Norte; Alfa Kenia Fernández, Hospital Metropolitano de Santiago (HOMS); Licurgo Jacob Cruz‐Díaz, Hospital Universitario Presidente Estrella Ureña.

**Suriname:** Kwame van der Hilst, Academic Hospital Paramaribo.

**Uruguay:** Gabriela Ormaechea‐Gorricho, Hospital Clínicas Doctor Manuel Quintela; Maximiliano Pereda, Médica Uruguaya.

**Venezuela:** Raquel E González‐Hormostay, Instituto Ascardio; Ximena Patricia Madrid Viteri, Clínica Chilemex; Luis Díaz Espinoza, Centro Clínico Santa Rosa; Eglee Castillo González, Centro Policlínico Valencia; Elirub Rojas Gimon, Cardiología Tropical Anzoátegui; Maite Alexandra González Fuentes, Hospital Universitario de Los Andes; Antonella A. Ferrer, Unidad Cardiovascular Ferrer; Gerardo Chazzin, UNICOR/FUNVIC; Niurka Pereira Velasco, IVSS Hospital Dr Rafael Calles Sierra; José Jesús Marval Saud, ATIMED C.A.

## Sources of Funding

AstraZeneca helped finance the development of a digital tool for the identification, recognition, and reporting of queries and inconsistencies in the Research Electronic Data Capture database.

## Disclosures

None.

## Supporting information

Tables S1–S4

STROBE Checklist
