# Supplementary material for: AMERICCAASS Registry: A Contemporary View of Heart Failure in the Americas
Source: J Am Heart Assoc. 2026 Aug 27;15(17):e047844. doi: 10.1161/JAHA.125.047844 (PMC13695805; doi:10.1161/JAHA.125.047844)
Supplement: Supplementary file 1 [file JAH3-15-e047844-s001.pdf]

SUPPLEMENTAL MATERIAL

Table S1. Participating centers and patients by country.

Country	Centers (n)	Patients (n)	Patients (%)
Cuba	17	1610	23.9
Colombia	17	1188	17.6
Argentina	16	603	9.0
Venezuela	10	478	7.1
Paraguay	8	450	6.7
Guatemala	5	351	5.2
Ecuador	10	306	4.5
Dominican Republic	4	294	4.4
Perú	6	268	4.0
México	7	165	2.5
Costa Rica	3	156	2.3
El Salvador	3	152	2.3
Uruguay	2	144	2.1
United States	2	137	2.0
Bolivia	2	129	1.9
Suriname	1	104	1.5
Jamaica	1	90	1.3
Panamá	1	62	0.9
Honduras	2	23	0.3
Nicaragua	1	21	0.3
Chile	1	1	0.0

Table S2. Complete etiologies

Variable	Overall, N = 6732	Inpatient, n = 2029	Outpatient, n = 4703
Ischemic, n (%)	2639 (39.9)	728 (35.9)	1911 (40.6)
Hypertensive, n (%)	1207 (17.9)	356 (17.5)	851 (18.1)
Valvular, n (%)	645 (9.6)	245 (12.1)	400 (8.5)
Idiopathic, n (%)	627 (9.3)	161 (7.9)	466 (9.9)
Tachycardiomyopathy, n (%)	350 (5.2)	95 (4.7)	255 (5.4)
Chagas, n (%)	227 (3.4)	89 (4.4)	138 (2.9)
Diabetes mellitus, n (%)	138 (2.0)	48 (2.4)	90 (1.9)
Viral, n (%)	99 (1.5)	28 (1.4)	71 (1.5)
Chemotherapy, n (%)	93 (1.4)	27 (1.3)	66 (1.4)
Congenital, n (%)	85 (1.3)	25 (1.2)	60 (1.3)
Alcoholic, n (%)	69 (1.0)	27 (1.3)	42 (0.9)
Other, n (%)	553 (8.2)	200 (9.9)	353 (7.5)

Table S3. Baseline characteristics according to LVEF data availability

Variable	Inpatient, n = 2029			Outpatient, n = 4703		
	LVEF available, n = 1501	LVEF missing, n = 528	p value	LVEF available, n = 4092	LVEF missing, n = 611	p value
Age (years), median (IQR)	67.5 (57.8, 78.1)	69.4 (58.6, 78.9)	0.2	68.7 (59.0, 77.3)	68.7 (58.8, 78.0)	0.6
Sex distribution*			0.024			0.8
Males, n (%)	922 (61.4)	294 (55.7)		1739 (42.5)	264 (43.2)	
Females, n (%)	579 (38.6)	234 (44.3)		2350 (57.5)	347 (56.8)	
Subregion			<0.001			<0.001
North and Central America, n (%)	142 (9.5)	117 (22.2)		766 (18.7)	42 (6.9)	
Caribbean, n (%)	402 (26.8)	166 (31.4)		934 (22.8)	492 (80.5)	
South America, n (%)	957 (63.8)	245 (46.4)		2392 (58.5)	77 (12.6)	
History of HF			<0.001			<0.001
New onset, n (%)	462 (30.8)	302 (57.2)		1229 (30.0)	284 (46.5)	
Chronic, n (%)	1039 (69.2)	226 (42.8)		2863 (70.0)	327 (53.5)	
Baseline medical history						
Hypertension, n (%)	1101 (73.4)	320 (60.6)	<0.001	2905 (71.0)	361 (59.1)	<0.001
Dyslipidemia, n (%)	598 (39.8)	108 (20.5)	<0.001	1955 (47.8)	141 (23.1)	<0.001
Coronary artery disease, n (%)	551 (36.7)	143 (27.1)	<0.001	1745 (42.6)	201 (32.9)	<0.001

Diabetes, n (%)	459 (30.6)	165 (31.3)	0.8	1151 (28.1)	126 (20.6)	<0.001
Atrial fibrillation, n (%)	459 (30.6)	93 (17.6)	<0.001	1087 (26.6)	103 (16.9)	<0.001
NYHA			0.019			0.059
Class I, n (%)	37 (2.5)	11 (2.3)		818 (20.3)	109 (24.2)	
Class II, n (%)	311 (20.8)	129 (26.9)		2113 (52.5)	243 (53.9)	
Class III, n (%)	700 (46.9)	192 (40.0)		984 (24.4)	90 (20.0)	
Class IV, n (%)	445 (29.8)	148 (30.8)		113 (2.8)	9 (2.0)	
Unknown	8 (0.5)	48 (9.1)		64 (1.6)	160 (26.2)	

Abbreviations: HF: Heart failure; IQR: Interquartile range; LVEF: Left ventricular ejection fraction; NYHA: New York Heart Association.

** Sex was available in 4,089 of 4,092 outpatients with available LVEF; missing data: 3 (0.1%).*

Table S4. Baseline characteristics by region (North/Central America, the Caribbean, and South America).

Variable	Overall, N = 6732	Caribbean, n = 1994	North and Central America, n = 1067	South America, n = 3671
Age (years), median (IQR)	68.6 (58.8, 77.7)	68.8 (59.0, 77.9)	66.5 (56.0, 75.9)	69.0 (59.3, 78.1)
Sex distribution*				
Males, n (%)	3913 (58.2)	1138 (57.2)	594 (55.7)	2181 (59.4)
Females, n (%)	2816 (41.8)	853 (42.8)	473 (44.3)	1490 (40.6)
History of HF				
New onset, n (%)	2277 (33.8)	727 (36.5)	413 (38.7)	1137 (31.0)
Chronic, n (%)	4455 (66.2)	1267 (63.5)	654 (61.3)	2534 (69.0)
Baseline medical history				
Hypertension, n (%)	4687 (69.6)	1344 (67.4)	646 (60.5)	2697 (73.5)
Dyslipidemia, n (%)	2802 (41.6)	454 (22.8)	429 (40.2)	1919 (52.3)
Coronary artery disease, n (%)	2640 (39.2)	858 (43)	367 (34.4)	1415 (38.5)
Diabetes, n (%)	1901 (28.2)	444 (22.3)	391 (36.6)	1066 (29)
Atrial fibrillation, n (%)	1742 (25.9)	402 (20.2)	247 (23.1)	1093 (29.8)
LVEF*				
≤ 40, n (%)	3105 (55.5)	622 (46.6)	604 (66.5)	1879 (56.1)
41-49, n (%)	934 (16.7)	289 (21.6)	114 (12.6)	531 (15.9)
≥ 50, n (%)	1554 (27.8)	425 (31.8)	190 (20.9)	939 (28.0)
Unknown	1139 (16.9)	658 (33)	159 (14.9)	322 (8.8)
NYHA				
Class I, n (%)	975 (15.1)	159 (8.8)	202 (19.2)	614 (17)
Class II, n (%)	2796 (43.3)	861 (47.9)	353 (33.6)	1582 (43.9)
Class III, n (%)	1966 (30.5)	647 (36.0)	331 (31.5)	988 (27.4)
Class IV, n (%)	715 (11.1)	132 (7.3)	164 (15.6)	419 (11.6)
Unknown	280 (4.2)	195 (9.8)	17 (1.6)	68 (1.9)

Abbreviations: HF: Heart failure; IQR: Interquartile range; LVEF: Left ventricular ejection fraction; NYHA: New York Heart Association.

Patients were grouped according to the region of the participating institutions as follows: Caribbean (n = 1,994; 29.6%), North and Central America (n = 1,067; 15.9%), and South America (n = 3,671; 54.5%). Countries included in each region were Cuba, Dominican Republic, and Jamaica for the Caribbean; United States, Mexico, Costa Rica, El Salvador, Guatemala, Honduras, Nicaragua, and Panama for North and Central America; and Argentina, Bolivia, Chile, Colombia, Ecuador, Paraguay, Peru, Suriname, Uruguay, and Venezuela for South America. North and Central America were analyzed jointly because of sample size considerations.

** Sex was available in 6,729 patients (99.9%); missing data: 3 overall (<0.1%), all in the Caribbean subgroup*
