# Supplementary material for: AMERICCAASS Registry: A Contemporary View of Heart Failure in the Americas
Source: J Am Heart Assoc. 2026 Aug 27;15(17):e047844. doi: 10.1161/JAHA.125.047844 (PMC13695805; doi:10.1161/JAHA.125.047844)
Supplement: Supplementary file 2 [file JAH3-15-e047844-s002.pdf]

STROBE Statement—checklist of items that should be included in reports of observational studies

	Item No.	Recommendation	Page No.	Relevant text from manuscript
**Title and abstract**	1	(*a*) Indicate the study's design with a commonly used term in the title or the abstract	1, 4	*Title: The AMERICCAASS Registry: A Contemporary View of Heart Failure in the Americas / Abstract: Methods: The AMERICCAASS Registry is a prospective, multicenter, observational study involving 119 institutions across 21 countries in the Americas, including adults with HF enrolled as inpatients or outpatients. Sociodemographic and clinical data were analyzed descriptively.*
		(*b*) Provide in the abstract an informative and balanced summary of what was done and what was found	4	*Abstract: Methods: The AMERICCAASS Registry is a prospective, multicenter, observational study involving 119 institutions across 21 countries in the Americas, including adults with HF enrolled as inpatients or outpatients. Sociodemographic and clinical data were analyzed descriptively. Results: A total of 6,732 patients were included; 30.1% were inpatients and 69.9% outpatients. The median age was 68 years, and 58.2% were male. Hypertension (69.6%), dyslipidemia (41.6%), and coronary artery disease (39.2%) were the most common comorbidities. Ischemic (39.9%) and hypertensive (17.9%) etiologies predominated, while Chagas disease was present in 3.4%. Unhealthy habits were common, with 39.8% reporting smoking, 24.4% alcohol use, and only 23.9% regular physical activity. New York Heart Association (NYHA) class III predominated in inpatients (45.2%) whereas NYHA class II predominated in outpatients (52.6%). HF with reduced left ventricular ejection fraction (HFrEF) was the most common phenotype in both settings: inpatients (56.8%) and outpatients (55.1%). Across settings, beta-blockers and mineralocorticoid receptor antagonists were the most frequently prescribed therapies, followed by sodium–glucose cotransporter-2 inhibitors, whereas ACE inhibitors, ARNIs, and ARBs were less commonly used. Among inpatients, intensive care unit (ICU) admission occurred in 55.9% and in-hospital mortality was 7%.*
**Introduction**				
Background/rationale	2	Explain the scientific background and rationale for the investigation being reported	5 - 6	*Heart failure (HF) is a significant health challenge worldwide due to its high prevalence, substantial morbidity and mortality, and the increasing burden it places on healthcare systems / Despite this distinctive epidemiological profile and the fact that these countries bear approximately 80% of the global cardiovascular disease burden, the majority of HF research continues to originate from the US and Europe 7. Historically, cardiovascular research output has been concentrated in these regions, with more limited representation from Latin America (LATAM)*
Objectives	3	State specific objectives, including any prespecified hypotheses	6	*The AMERICCAASS Registry (Registro Americano de Insuficiencia Cardiaca Ambulatoria o Agudamente Descompensada) was established to address gaps in the epidemiological and*

				clinical understanding of HF in the region. Its primary objective is to generate robust multicenter data that more accurately reflect the reality of HF in the Americas, thereby strengthening the characterization of sociodemographic patterns, risk factors, clinical progression, and patient outcomes across the continent
**Methods**				
Study design	4	Present key elements of study design early in the paper	6 - 7	*The AMERICCAASS Registry is a multicenter, observational, and prospective study involving healthcare institutions across North, Central, South America, and the Caribbean.*
Setting	5	Describe the setting, locations, and relevant dates, including periods of recruitment, exposure, follow-up, and data collection	6 - 7	*Participating centers were healthcare institutions from countries affiliated with the Sociedad Interamericana de Cardiología (SIAC). Site participation was established through an open invitation disseminated via the SIAC network, and enrollment was voluntary. / Recruitment began in April 2022 and concluded in November 2023.*
Participants	6	(*a*) *Cohort study*—Give the eligibility criteria, and the sources and methods of selection of participants. Describe methods of follow-up *Case-control study*—Give the eligibility criteria, and the sources and methods of case ascertainment and control selection. Give the rationale for the choice of cases and controls *Cross-sectional study*—Give the eligibility criteria, and the sources and methods of selection of participants	7 - 8	*Individuals aged ≥18 years with a confirmed HF diagnosis, either in the inpatient or outpatient setting, were included. HF diagnosis was established based on documentation in medical records and PI evaluation. Inclusion and exclusion criteria are summarized in Table 1.*
		(*b*) *Cohort study*—For matched studies, give matching criteria and number of exposed and unexposed *Case-control study*—For matched studies, give matching criteria and the number of controls per case		Not applicable.
Variables	7	Clearly define all outcomes, exposures, predictors, potential confounders, and effect modifiers. Give diagnostic criteria, if applicable	7 - 8	*Information was collected for each participant from electronic medical records corresponding to outpatient visits or hospital admissions. Sociodemographic data, including self-reported race, comorbidities, medical history, pre-admission medication use, lifestyle habits, and the most recent left ventricular ejection fraction (LVEF) prior to evaluation were recorded.* *Lifestyle habits included smoking history, alcohol consumption, and physical activity. Smoking history was based on the presence of any past or current tobacco use as documented in the clinical history. Alcohol consumption was assessed based on frequency. Physical*

				activity was recorded as the number of days per week in which the patient reported performing any kind of exercise, irrespective of intensity or duration. The New York Heart Association (NYHA) functional class was assessed at hospital admission for inpatients and during the clinical visit for outpatients.<br>*In inpatients, additional clinical information was collected, including the etiology of HF based on prior history and diagnostic workup, the primary cause of the acute decompensation episode, the clinical presentation at admission, the hemodynamic profile according to clinical assessment, and the patient's condition at discharge. Pharmacological management at hospital discharge or therapeutic plans established during outpatient visits were also documented.*
Data sources/ measurement	8*	For each variable of interest, give sources of data and details of methods of assessment (measurement). Describe comparability of assessment methods if there is more than one group	7–8	*Information was collected for each participant from electronic medical records corresponding to outpatient visits or hospital admissions / Participant information was stored on Research Electronic Data Capture (REDCap) platform to ensure anonymity, confidentiality, and data security. / To comprehensively characterize the study population, descriptive statistical analyses were performed on sociodemographic and clinical variables. The distribution of quantitative variables was assessed using the Shapiro–Wilk and Kolmogorov–Smirnov tests to evaluate normality. Continuous variables were summarized as medians and interquartile ranges for non-normal distributions, or as means and standard deviations when distributions approximated normality. Categorical variables were presented as absolute frequencies and percentages.Missingness was assessed across all variables, with most showing <1% missingness. Relevant missing data are reported, and variables were summarized using available observations. For analyses using LVEF-based HF phenotypes, only patients with complete LVEF data were included.*
Bias	9	Describe any efforts to address potential sources of bias	6 - 8, 17	Potential sources of bias were addressed through standardized electronic data capture, PI review of HF diagnosis, and discussion of limitations including network-based sampling, self-reported variables, and variability in documentation practices.
Study size	10	Explain how the study size was arrived at	9	All eligible patients recruited during the study period were included. Recruitment ran from April 2022 to November 2023. Of 6,798 patients in the original dataset, 66 were excluded for data quality/eligibility reasons, leaving 6,732 for final analysis.

Continued on next page

Quantitative variables	11	Explain how quantitative variables were handled in the analyses. If applicable, describe which groupings were chosen and why	8	*Continuous variables were summarized as medians and interquartile ranges for non-normal distributions, or as means and standard deviations when distributions approximated normality. Categorical variables were presented as absolute frequencies and percentages.*
Statistical methods	12	(*a*) Describe all statistical methods, including those used to control for confounding	8	*To comprehensively characterize the study population, descriptive statistical analyses were performed on sociodemographic and clinical variables. The distribution of quantitative variables was assessed using the Shapiro–Wilk and Kolmogorov–Smirnov tests to evaluate normality / Missingness was assessed across all variables, with most showing <1% missingness. Relevant missing data are reported, and variables were summarized using available observations. For analyses using LVEF-based HF phenotypes, only patients with complete LVEF data were included.*<br><br>Descriptive statistical analyses were performed on sociodemographic and clinical variables. No multivariable analyses or statistical adjustment for confounding were performed.
		(*b*) Describe any methods used to examine subgroups and interactions	8, 10 - 11	Additional analyses included stratification by care setting and by LVEF group for medication prescription patterns.
		(*c*) Explain how missing data were addressed	8	*Missingness was assessed across all variables, with most showing <1% missingness. Relevant missing data are reported, and variables were summarized using available observations. For analyses using LVEF-based HF phenotypes, only patients with complete LVEF data were included.*
		(*d*) *Cohort study*—If applicable, explain how loss to follow-up was addressed<br>*Case-control study*—If applicable, explain how matching of cases and controls was addressed<br>*Cross-sectional study*—If applicable, describe analytical methods taking account of sampling strategy		Not applicable; no longitudinal follow-up or loss-to-follow-up analysis was performed in this descriptive registry report beyond the index hospitalization/outpatient encounter
		(*e*) Describe any sensitivity analyses	Supplementary material (Table 3)	A sensitivity analysis was performed by comparing baseline characteristics between patients with and without available LVEF data in both inpatient and outpatient settings

Results

Participants	13*	(a) Report numbers of individuals at each stage of study—eg numbers potentially eligible, examined for eligibility, confirmed eligible, included in the study, completing follow-up, and analysed	9	*Of 6,798 patients in the original dataset, 66 were excluded for data quality/eligibility reasons. Thus, 6,732 patients were considered for the final analysis (Figure 1). These patients were enrolled at 119 medical institutions across 21 countries in the Americas (Figure 2). / Among these, 2,029 patients (30.1%) were inpatients, and 4,703 (69.9%) were outpatients.*
		(b) Give reasons for non-participation at each stage	9	*Of 6,798 patients in the original dataset, 66 were excluded for data quality/eligibility reasons. Thus, 6,732 patients were considered for the final analysis (Figure 1).*
		(c) Consider use of a flow diagram	9	*Figure 1. Study flow diagram and final analytic sample*
Descriptive data	14*	(a) Give characteristics of study participants (eg demographic, clinical, social) and information on exposures and potential confounders	9 -11	Participant characteristics, including age, sex, ethnicity, HF history, comorbidities, lifestyle habits, LVEF, NYHA class, causes of decompensation, clinical presentation, hemodynamic profile, ICU admission, and discharge condition, are presented in the Results and shown in Tables 2 and 3.
		(b) Indicate number of participants with missing data for each variable of interest	9 -11	Missing data were reported in the text and in table footnotes, including for age, sex, ethnicity, LVEF, NYHA class, clinical presentation, hemodynamic profile, ICU admission, and discharge condition.
		(c) *Cohort study*—Summarise follow-up time (eg, average and total amount)		Not applicable; no follow-up time was analyzed in this report.
Outcome data	15*	*Cohort study*—Report numbers of outcome events or summary measures over time	*10*	For inpatients, outcome data included ICU admission (55.9%) and in-hospital mortality (7%).
		Case-control study—Report numbers in each exposure category, or summary measures of exposure		Not applicable
		Cross-sectional study—Report numbers of outcome events or summary measures		Not applicable
Main results	16	(*a*) Give unadjusted estimates and, if applicable, confounder-adjusted estimates and their precision (eg, 95% confidence interval). Make clear which confounders were adjusted for and why they were included		Not applicable; this study presents descriptive summary measures rather than unadjusted/adjusted effect estimates with confidence intervals.
		(*b*) Report category boundaries when continuous variables were categorized	9 - 10	Category boundaries were reported for grouped continuous variables, including age categories **and** LVEF categories (≤40, 41–49, ≥50)**.**
		(*c*) If relevant, consider translating estimates of relative risk into absolute risk for a meaningful time period		Not applicable; no relative risk estimates were reported.

Continued on next page

Other analyses	17	Report other analyses done—eg analyses of subgroups and interactions, and sensitivity analyses	10 -11	*Other analyses included medication prescription patterns stratified by care setting and by LVEF group, with detailed percentages shown in Figures 4–6*
**Discussion**				
Key results	18	Summarise key results with reference to study objectives	11	*The AMERICCAASS Registry represents the first collaborative effort to characterize HF across multiple countries in the Americas. Our findings confirm that HF remains a major health challenge in the region, with several critical patterns emerging from the data. Notably, we observed high rates of unhealthy lifestyle habits, suboptimal use of GDMT, and a high prevalence of HFrEF. ICU admission rates were strikingly high, and in-hospital mortality remained considerable. These findings highlight regional challenges and support context-specific interventions to improve HF outcomes across the Americas.*
Limitations	19	Discuss limitations of the study, taking into account sources of potential bias or imprecision. Discuss both direction and magnitude of any potential bias	17	Limitations included the descriptive design, possible underrepresentation of lower-resource regions, network-based sample, absence of Brazil, possible variability in HF diagnosis based on PI judgment, self-reported data, and documentation-related missingness.
Interpretation	20	Give a cautious overall interpretation of results considering objectives, limitations, multiplicity of analyses, results from similar studies, and other relevant evidence	11 - 17	The Discussion provides an overall interpretation of findings in light of previous literature, clinical severity, regional challenges, and treatment gaps across the Americas.
Generalisability	21	Discuss the generalisability (external validity) of the study results	17	*Regions with limited resources and restricted healthcare access may be underrepresented, potentially biasing the dataset toward better-resourced settings and affecting the generalizability of the findings. Although the AMERICCAASS Registry provides broad geographic coverage through the SIAC network, participation followed an open invitation and voluntary enrollment; therefore, it represents a network-based sample that may not be nationally representative within each participating country nor fully representative of the broader Americas region. Notably, the absence of Brazil, a key contributor to cardiovascular research in the Americas, may limit the applicability of our findings, particularly for conditions such as Chagas disease.*
**Other information**				
Funding	22	Give the source of funding and the role of the funders for the present study and, if applicable, for the original study on which the present article is based	18	*This research received no specific grant from any funding agency in the public, commercial, or not-for-profit sectors, and did not receive any financial support from industry.*

*Give information separately for cases and controls in case-control studies and, if applicable, for exposed and unexposed groups in cohort and cross-sectional studies.

Note: An Explanation and Elaboration article discusses each checklist item and gives methodological background and published examples of transparent reporting. The STROBE checklist is best used in conjunction with this article (freely available on the Web sites of PLoS Medicine at http://www.plosmedicine.org/, Annals of Internal Medicine at http://www.annals.org/, and Epidemiology at http://www.epidem.com/). Information on the STROBE Initiative is available at www.strobe-statement.org.
